# Initiation of mRNA decay in bacteria

**DOI:** 10.1007/s00018-013-1472-4

**Published:** 2013-09-25

**Authors:** Soumaya Laalami, Léna Zig, Harald Putzer

**Affiliations:** CNRS UPR9073 (Associated with Université Paris Diderot, Sorbonne Paris Cité), Institut de Biologie Physico-Chimique, 13-rue Pierre et Marie Curie, 75005 Paris, France

**Keywords:** mRNA degradation, RNase E, RNase J, RNase Y, Gene expression, Prokaryote

## Abstract

The instability of messenger RNA is fundamental to the control of gene expression. In bacteria, mRNA degradation generally follows an “all-or-none” pattern. This implies that if control is to be efficient, it must occur at the initiating (and presumably rate-limiting) step of the degradation process. Studies of *E. coli* and *B. subtilis*, species separated by 3 billion years of evolution, have revealed the principal and very disparate enzymes involved in this process in the two organisms. The early view that mRNA decay in these two model organisms is radically different has given way to new models that can be resumed by “different enzymes—similar strategies”. The recent characterization of key ribonucleases sheds light on an impressive case of convergent evolution that illustrates that the surprisingly similar functions of these totally unrelated enzymes are of general importance to RNA metabolism in bacteria. We now know that the major mRNA decay pathways initiate with an endonucleolytic cleavage in *E. coli* and *B. subtilis* and probably in many of the currently known bacteria for which these organisms are considered representative. We will discuss here the different pathways of eubacterial mRNA decay, describe the major players and summarize the events that can precede and/or favor nucleolytic inactivation of a mRNA, notably the role of the 5′ end and translation initiation. Finally, we will discuss the role of subcellular compartmentalization of transcription, translation, and the RNA degradation machinery.

## Introduction

Messenger RNA (mRNA) is short-lived. In bacteria, the half-lives of mRNAs can vary from seconds to over an hour, but they are generally much shorter than the doubling time of the organism. This metabolic instability is crucial for (1) adapting the pattern of gene expression to a changing environment, which is often controlled at the level of transcription, (2) producing the correct amount of a given protein, and (3) recycling of ribonucleotides for incorporation into new RNA molecules.

For all of these reasons, mRNA degradation must be precisely controlled, notably to maximize the competitivity of bacteria in a possibly hostile environment. The only efficient way to regulate mRNA decay is to control the steps initiating degradation. Indeed, mRNA decay in bacteria generally follows first-order kinetics, depending on a rate-determining initial step. Decay intermediates are rarely observed, i.e., Northern analysis of a particular mRNA generally reveals the full-length transcript. This all-or-none pattern is typical for all bacterial species studied to date. For example, after an mRNA suffers a first endonucleolytic cleavage, the scavenging process is so rapidly initiated that the resulting fragments are usually not detected, unless one or more ribonucleases involved in this process is inactivated. However, not all cleavages are synonymous with degradation. Indeed, in some cases, a transcript can also be “processed”, i.e., the major translated species found in the cell in vivo is not the primary transcript. Notably, the processing of polycistronic transcripts allows uncoupling the expression of various proteins encoded within an operon, a phenomenon widely observed in prokaryotes (e.g., [1–4]).

The lifetime of each mRNA species is unique. What we commonly refer to as “stability” is the chemical lifetime of an RNA. This is the period during which the original full-size transcript remains physically intact. The moment of initial nucleolytic inactivation of an mRNA, which impairs its translation, is determined by a variety of parameters. These include translation efficiency, RNA sequence and secondary structure, the interaction with proteins or other RNAs, and possibly also the subcellular location. This implies that non-nucleolytic events can influence the “functional” lifetime of an mRNA, i.e., the time during which it can support protein synthesis. While the functional lifetime can obviously not exceed the chemical lifetime of an mRNA, it can be shorter [5, 6]. For example, the tight binding of a translational repressor that blocks translation initiation by competing with the ribosome for access to the Shine-Dalgarno sequence (non-nucleolytic inactivation) can lead to the immediate destruction of the mRNA [7]. *Trans*-encoded small regulatory RNAs (sRNA) can have a similar decay-initiating effect by binding to the RBS region of an mRNA, thereby repressing translation. Expression of these regulatory sRNAs is generally in response to a stress condition [8, 9]. In these cases, initiation of mRNA degradation is secondary to translational repression, but the two processes probably occur near simultaneously in most cases [5]. Only very few studies have addressed the global importance of non-nucleolytic inactivation of mRNA under steady-state growth conditions. In one of them, it was shown that removing the C-terminal half of the key endoribonuclease E in *E. coli* does not impair logarithmic growth and increases the functional and physical life-times of bulk mRNA alike by about twofold [10, 11]. Similarly, depletion of RNase E leading to slower but still exponential growth causes a twofold increase in the functional half-life of bulk mRNA [12]. This implies that at least in *E. coli* nucleolytic inactivation is the dominant path to functional inactivation of an mRNA.

In bacteria, the chemical stability of mRNAs does not appear to be correlated with or proportional to the doubling time. For fast-growing bacteria (doubling time <1 h), the average half-lives of bulk mRNA are in the range of 2–10 min. Some variation can also be due to experimental differences (e.g., diverse strains, whether measured at 30 or 37 °C): 2.1–6.8 min in *E. coli* [10, 13–15], 2.6–5 min in *B. subtilis* [16–18], <5 min for 90 % of log phase mRNAs in *Staphylococcus aureus* [19], ~1 min in *Streptococcus pyogenes* [20] and from 6 min (exponential growth) to 19 min (under glucose starvation) in *Lactococcus lactis* [21]. A similar bulk mRNA half-life (5.2 min) was found in *Mycobacterium smegmatis* (doubling time = 2–3 h) but a somewhat longer mean half-life for log phase transcripts (9.5 min) was observed in *Mycobacterium tuberculosis*, which has a doubling time of about 20 h [22]. On the other hand, the marine cyanobacterium *Prochlorococcus* that also divides only about once a day has an average mRNA half-life of only 2.4 min [23]. Under laboratory growth conditions, all known bacterial mRNA turnover rates are thus quite fast but also disparate with respect to their growth rate. This likely reflects evolutionary adaptation of each organism to its environment.

Theoretically, there are three ways to initiate nucleolytic decay of an mRNA: exonucleolytical attack of the ends (5′ or 3′) and endonucleolytic cleavage within the body of the message. All known bacteria have 3′ exoribonucleases, but they are likely not used to degrade mRNA from the 3′ end on a large scale (see below). Indeed, this would be a biologically inefficient and wasteful process that accumulates incomplete polypeptides from truncated mRNAs. The mechanisms by which transcripts are degraded obviously depend on the enzymes available in a given organism. Interestingly, the major ribonucleases involved in the initiation of mRNA decay in the two model organisms *E. coli* and *B. subtilis* are very different [24]. Recent progress in the characterization of novel ribonucleases (notably RNases J and Y) from different organisms suggests that the presence of particular enzymes is not synonymous with different strategies for initiating mRNA degradation.

In the first part of this review, we will discuss unexpected similarities between major ribonucleases, which are completely unrelated at the protein sequence, and the substantial evidence accumulating in favor of internal cleavage of an mRNA as being the major pathway to start degrading a transcript. Secondly, we summarize the events that can precede and/or favor nucleolytic inactivation of a mRNA, notably the role of the 5′ end and translation initiation. Finally, we will discuss the role of subcellular compartmentalization of transcription, translation, and the RNA degradation machinery.

## Disparate enzymes and convergent evolution

Cleavage within the body of a transcript is a very efficient and definitive way to inactivate an mRNA and initiate its decay. In this process, the primordial role of endoribonucleases with relaxed sequence specificity that produces short-lived decay intermediates is now clearly recognized. The founding member of this class of ribonucleases is RNase E [25–29]. In *E. coli*, under steady-state growth conditions, the decay of most mRNAs begins with an internal cleavage by the essential RNase E [30]. In accordance, heat inactivation of a thermosensitive RNase E mutant increased the chemical stability of bulk mRNA up to fivefold, from about 2.5 min to over 10 min [13, 28]. RNase E does not depend on a particular nucleotide sequence for cleavage but requires a single-stranded region preferably rich in AU residues. RNase E cleavages are nevertheless quite specific, in vivo and in vitro, presumably because of structural constraints and other parameters that are still poorly understood [31–35]. Occasionally, mRNA decay in *E. coli* has been shown to involve other more specialized endoribonucleases. They include RNase G, a non-essential paralog of RNase E [36, 37], RNase III [38–41], RNase P [42, 43], RNase LS [44], RNase Z (BN) [45, 46], and maybe RNase H [47]. In particular, the role of RNase III in RNA metabolism has been studied in a variety of other organisms, notably *B. subtilis* and *S. aureus.* Even though this enzyme is essential in *B. subtilis* [48] due to its role in silencing of prophage-encoded toxin genes [49], the number of direct mRNA substrates appears to be rather limited [50] compared to more globally acting decay initiating enzymes like RNase E. In *S. aureus*, RNase III might play a more important role by assuming global regulatory functions in gene expression and might affect the turnover of structured mRNAs [51, 52] (see below).

Despite its crucial role in mRNA decay, many bacterial species like the Gram-positive model organism *B. subtilis*, do not contain an RNase E [53]. The large evolutionary distance between *E. coli* and *B. subtilis* (about 3 billion years, [54]) turned out to be very beneficial for the analysis of bacterial mRNA metabolism. The advent of routine genome sequencing confirmed the absence of particular ribonucleases, like RNase E, in certain classes of bacteria and thus led to the identification of new enzymes in these species. As the differences in the arsenal of ribonucleases in different species, particularly between Gram-negative and Gram-positive organisms, was confirmed, a kind of dogma gained acceptance that the overall mechanisms of RNA decay would also differ in these organisms. In *B. subtilis*, the stabilizing effect of 5′ “roadblocks” (e.g., a stalled ribosome) on long downstream regions of mRNA, even in the absence of translation, was a key observation that led to this idea [55].

Early studies on aminoacyl-tRNA synthetase genes regulated by tRNA-mediated antitermination in *B. subtilis* had shown that processing in an AU-rich region of the untranslated leader sequence was dependent on RNase E when the gene was expressed in *E. coli*. Since cleavage occurred at the same site in *B. subtilis*, it was suggested that an RNase E-like activity should also exist in *B. subtilis* [56]. It was only several years later that such an activity could be traced to a ribosome-associated fraction which, after purification, led to the identification of two paralogous ribonucleases now called RNases J1 (*rnjA*) and J2 (*rnjB*) encoded by genes of previously unknown function [16]. In addition to its RNase E-like endonucleolytic activity, RNase J1 was later shown to also possess exonucleolytic activity with a 5′–3′ polarity [57], an activity unprecedented in bacteria. RNase J1 was the first ribonuclease shown to perform two enzymatic activities, using a single catalytic site [58]. This enzyme fitted well with the perception that mRNA decay in *B. subtilis* differs greatly from the model proposed for *E. coli.* Known bona fide endonucleolytic targets for RNase J1 are rare and remain difficult to identify [24]. In addition, the 5′ exonuclease activity of RNase J1 perfectly explained the stabilizing effect of 5′ “roadblocks” on long untranslated downstream regions of mRNA. However, depletion of RNase J1 in a strain also lacking RNase J2 only modestly increased bulk mRNA stability from 2.6 to 3.6 min and single mutants showed no effect [16]. This hinted at the possibility that RNase J1/J2 was not the major enzyme initiating mRNA decay in *B. subtilis.* Indeed, a novel endoribonuclease named RNase Y, which when depleted increased the half-life of bulk mRNA more than twofold, was recently characterized. It cleaves in AU-rich single-stranded regions close to secondary structures in vitro and in vivo [59]. RNase Y sites resemble those described for RNase E [60, 61], even though only a few sites have so far been identified [3, 59, 62].

**Fig. 1:**
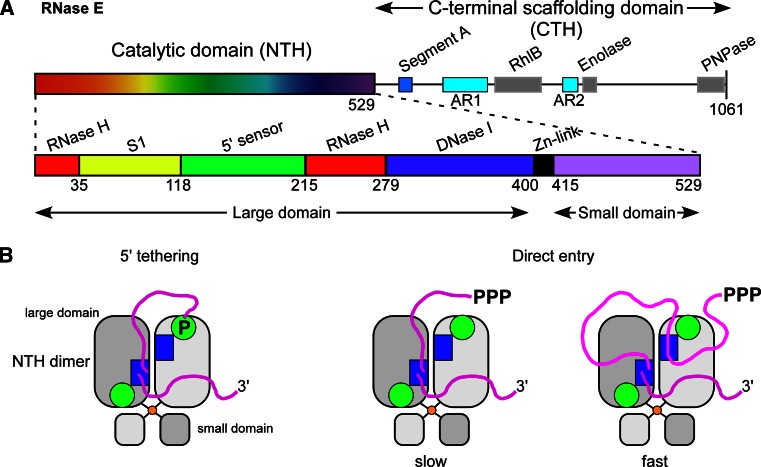
RNase E: domain structure and substrate binding. **a** Domain composition an RNase E monomer (1,061 aa). The catalytic amino-terminal half (NTH, aa 1–529) contains a large globular domain (aa 1–400), which is a composite of recurrent structural subdomains as shown [63] and a small folded domain (aa 415–529). The C-terminal half (CTH) of the protein is predicted to be unfolded but contains microdomains that mediate interactions with the cytoplasmic membrane (segment A) and other components of the degradosome (the helicase RhlB, enolase, and PNPase). AR1 and AR2 are arginine-rich segments probably involved in RNA binding. **b** RNase E exists primarily as a tetramer composed of a dimer of dimers [63]. The monomers of the principal dimer shown here are in light or dark grey and are held together by a dimer interface and a cooperatively coordinated Zn^+2^ ion (shown in *yellow*, the Zn-link, aa 400–415, [86]). Interactions between the small domains of the principal dimers stabilize the tetramer (not shown). Each protomer possesses a 5′ P binding pocket (*green circle*) and an active site (*blue rectangle*). In the 5′ tethering pathway, the monophosphorylated 5′ end of the mRNA (in *violet*) binds to the 5′ P binding pocket of one protomer, whereas cleavage occurs in the active site of the other protomer. The direct entry pathway that operates mainly on primary 5′ PPP transcripts is probably the major route for initiating mRNA decay in *E. coli,* but its efficiency is largely dependent on the conformation of the mRNA that is recognized by the nuclease. Binding of the substrate to only one active site is thought to be less efficient (slow) than, for example, the simultaneous binding of two single-stranded regions of which one might only serve to tether RNase E to the RNA (fast) [152]. However, binding of multiple sites should be very sensitive to ribosome occupancy and also be more demanding in terms of respecting enzyme geometry. Similarly, in the 5′ tethering pathway, RNase E preferentially cleaves sites, when available, in the 5′ UTR and avoids reaching around translating ribosomes [97]

**Fig. 2:**
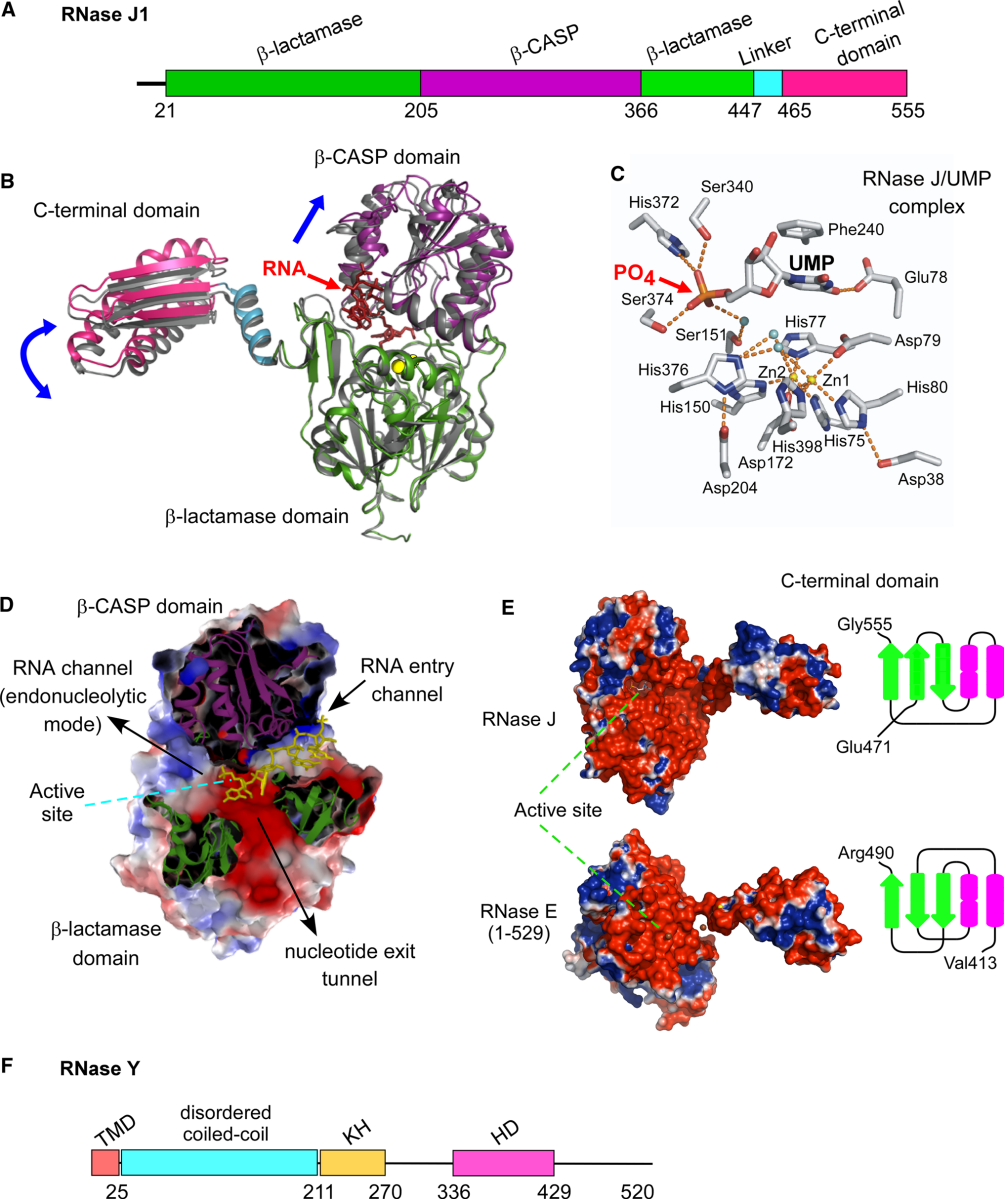
The architecture of RNases J and Y. **a** Domains composing *B. subtilis* RNase J1 (555 aa). The β-CASP domain is inserted into the β-lactamase domain to which the C-terminal domain is attached by a linker. **b** Comparison of the open and closed ribbon conformations of the *T. thermophilus* RNase J monomer. The open conformation is shown with colored backbone (in the presence of a 4 nt RNA, colored in *red*) [118, 119] and the closed free enzyme in *gray* [58]. The β-lactamase domain of the open conformation (in *green*) is superposed on that of the free enzyme to show the relative movements (*blue arrows*) of the β-CASP (in *violet*), C-terminal (in *pink*) and linker (in *blue*) domains. The catalytic Zn^+2^ ions in the active site are in *yellow*. **c** Close-up of the RNase J catalytic center complexed with an UMP residue. The 5′ terminal phosphate group is coordinated by serine and histidine residues in a phosphate binding pocket that provides a rationale for the enzyme’s requirement for a 5′ P in exonuclease mode [58]. *Dotted orange lines* indicate ligand-mediated and hydrogen bond interactions. **d** Slab view showing electrostatic surface predictions of the major RNase J domains (aa 1–447). Positively charged surfaces are shown in *blue* and negatively charged surfaces in *red*. The RNA is shown in *yellow*. The RNA-binding channel and a proposed nucleotide exit tunnel are indicated [118]. **e** Similar overall shape and electrostatic charge distribution between *T. thermophilus* RNase J and the catalytic N-terminal half of *E. coli* RNase E. The active site in both structures is facing upwards. The C-terminal domain of RNase J (aa 465–555) and RNase E (corresponding to the small domain in Fig. 1a, aa 415–529) share the same architecture, a three-stranded β-sheet facing two α-helices as shown. **f** Domains composing *B. subtilis* RNase Y (520 aa) include an N-terminal transmembrane domain (aa 1-25), followed by a large region predicted to be disordered (aa ~30–210), an RNA binding KH domain (aa 211–270) and a metal-chelating HD domain (aa 336–429) containing the conserved His/Asp motif required for RNase activity [59, 65, 166, 207]

So if there exist significant commonalities in the initiation of bacterial mRNA decay, they must derive in large part from the functions of the ribonucleases E, J, and Y. It is quite surprising to find three enzymes that can cleave mRNA with similar specificity. Indeed, RNases E, J, and Y show no similarity at the level of their primary sequence or in their mechanism of catalysis [24]. RNase E hydrolyses RNA via a DNase I-like domain [63] (Fig. 1a), RNase J activity relies on a β-CASP metallo-beta-lactamase fold [58, 64] (Fig. 2a, b) and RNase Y belongs to the HD family of metal-dependent phosphohydrolases [65] (Fig. 2f).

Nature has thus invented this endonucleolytic activity independently at least three times. However, it should be noted that the 3D structure of the catalytic N-terminal half of RNase E shows some surprising similarities with that of RNase J including a similar charge distribution [24, 58] and C-terminal domain architecture (Fig. 2e), but the real significance of this conservation remains enigmatic. This impressive case of convergent evolution illustrates that the functions of these enzymes are of general importance to mRNA metabolism in bacteria. In accordance, all prokaryotic *phyla* whose genomes have been sequenced contain at least one enzyme related to RNases E/G, J, or Y (Table 1). Moreover, all possible combinations of these enzymes in a single organism can be found. Some species rely on a single member like most of the β- and γ-proteobacteria, which almost exclusively have an RNase E/G type enzyme. Others, like many *Bacilli* (other than *B. subtilis)* and *Clostridium* or the δ-proteobacteria often have all three types of enzymes (Table 1). Outside the β- and γ-proteobacteria (e.g., in *Bacilli*), RNase E/G type enzymes are often short (less than 450 aa) corresponding in length to *E. coli* RNase G or the catalytic domain of RNase E. This implies that they can not form an *E. coli*-type degradosome (see below).

**Table 1:**
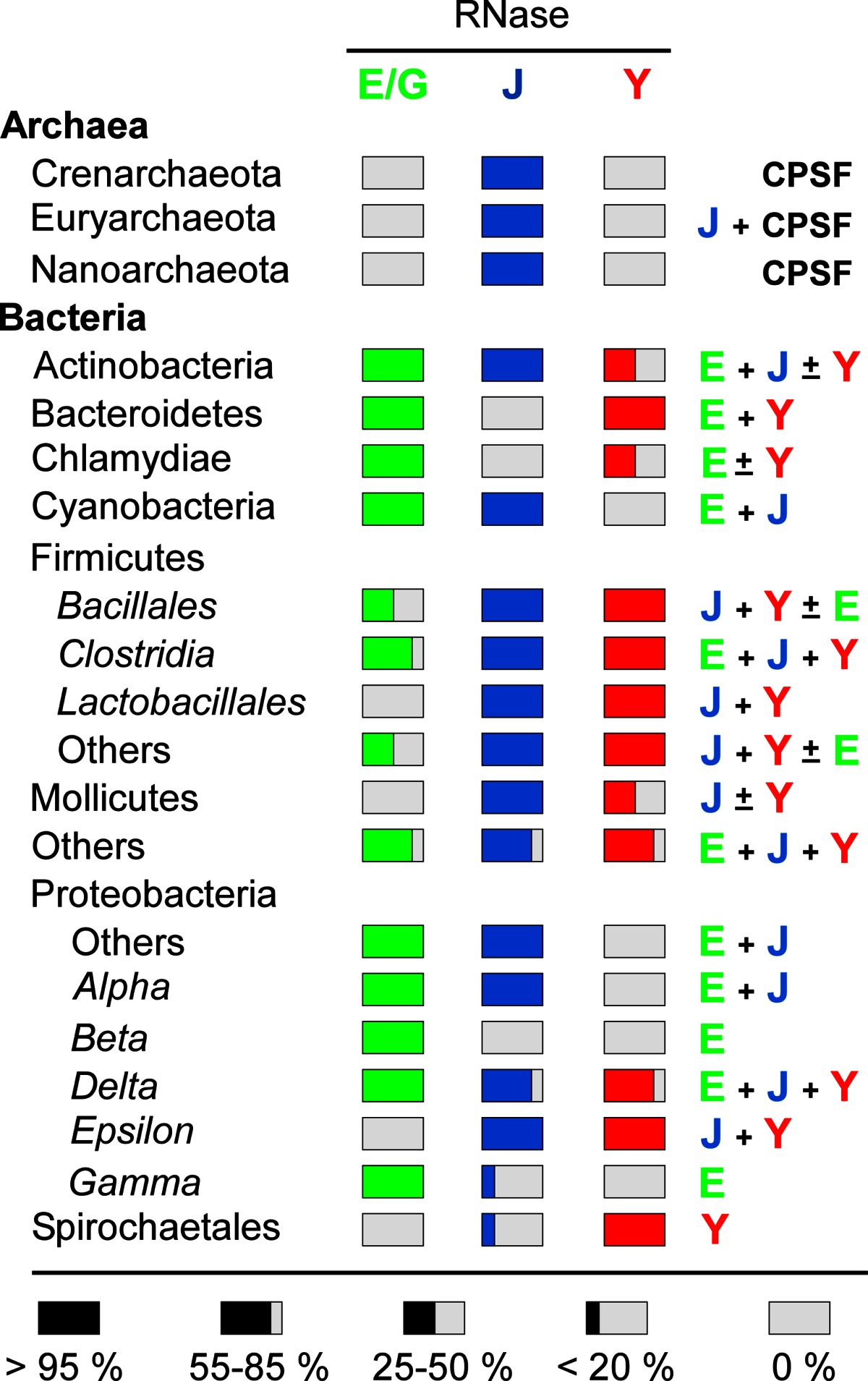
Occurrence of RNases E, J, and Y in prokaryotes

Percentage of species containing RNases E/G (green), J (blue) and/or Y (red) with respect to the total number of organisms within a *phylum*. Combinations of RNases present in the majority of a *phylum* are shown on the right. All Archaea contain an RNase J-like activity but they can be partitioned into two major subdivisions that correspond to orthologs of eukaryal cleavage and polyadenylation specificity factor (CPSF73) and bacterial RNase J [316]

We could thus look at these nucleases as a toolkit provided by evolution to adapt the strategies directing mRNA decay to individual needs. For example, in pathogenic Gram-positive bacteria, these nucleases have been implicated in the posttranscriptional control of a variety of genes that play important roles in virulence and biofilm formation (for a recent review see [66]).

The importance of an RNase (or any other enzyme for that matter) is often linked to its “essentiality” to support cell growth, generally measured in rich medium under laboratory conditions. Obviously, an enzyme is important if a cell cannot grow without it. However, the impact of the presence or absence of an enzyme can greatly vary in different organisms, under different circumstances and different growth conditions. A quick tour of the viability of species “surviving” the inactivation of the RNases E/G, J, or Y illustrates this. RNase E is required in *E. coli* for normal cell division [67] but no reason (i.e., specific targets) has yet been singled out for its essentiality [68–72]. In *Mycobacterium smegmatis*, which has an RNase E orthologue as well as RNase J only the former is essential [73]. Similarly, RNases J1 and J2 are both dispensable in *Staphylococcus aureus* ([74], P. Linder, pers. comm.) and *B. subtilis* [75]. In the latter, single cross inactivation of the *rnjA* gene, which produces a truncated RNase J1, is difficult to obtain, which is probably why RNase J1 was initially considered to be essential [76]. This contrasts with the situation in *Streptococcus pyogenes* where both paralogues RNases J1 and J2 are required for growth, as shown by the use of conditional mutants that only grow when induced [77]. RNase Y is not essential in *Streptococcus pyogenes* [78], *Staphylococcus aureus* [3], as well as in *B. subtilis* [75], where growth is nevertheless slowed considerably when the gene is absent.

The functional similarities between RNases E/G, J, and Y go well beyond a global effect on the transcript profile and a similar cleavage specificity. As we will describe below, they shed light on other parameters that are important for the initiation of mRNA degradation. These include a sensitivity concerning the nature of the mRNA 5′ end, a defined subcellular localization and a susceptibility to form multiprotein complexes called degradosomes.

Based essentially but not exclusively on studies in *E. coli* and *B. subtilis* we will summarize the major mRNA decay pathways identified in these organisms and how they depend on the characteristics of the major enzymes involved. The role of the mRNA 3′ end and tailing mechanisms (e.g., polyadenylation) in mRNA decay will only be described briefly here (“The 3′ end: tailing, scavenging and surveillance”), because 3′ exonucleolytic degradation does not play a major role in the initiation of mRNA decay. Our knowledge on the action of some of the decay-initiating ribonucleases is still very preliminary. Nevertheless, we will try to make the point that the RNases E, J, and Y should not be considered simply as doing the same job in any given organism. Instead, despite their surprising functional equivalence under certain circumstances, they allow for significant differences in the decay mechanisms that have been observed in various bacteria.

## The 5′ end: a target for exo- and endonucleases

The nature of the 5′ end of an mRNA can greatly influence transcript stability in bacteria. This appears obvious. Initiating the decay of a transcript from or near the 5′ end should rapidly lead to functional inactivation of the mRNA by removing any near-by RBS. At the same time, already engaged ribosomes can assure the translation of full-length proteins.

### Importance of the 5′ end for RNase E

In *E. coli*, the phosphorylation state of the 5′ end of an RNA has been shown to have a profound influence on its decay rate. This is due to the fact that RNase E, albeit an endoribonuclease, is sensitive to the nature of the substrate 5′ end. In vitro, it can cleave mRNA molecules much faster (>tenfold) when they carry an accessible 5′ P end instead of a 5′ PPP moiety, a base-paired 5′ P end or no 5′ end at all (circularized RNAs) [79].

RNase E (and its paralog RNase G) achieves this selectivity with the help of a discrete 5′ P binding pocket formed around Arg169, Thr 170 and Val128, that is distinct from its active site [63]. A comparison of the 3D structures of the holo- and apo-enzymes [80] revealed large conformational changes that occur during substrate binding. It is not immediately obvious how docking of the RNA 5′ P in the 5′ sensor domain would contribute to the conformational switch required for organizing the catalytic site. Although there is some debate in the literature on the precise role of the 5′ binding pocket, functionally, 5′ P docking in the 5′ sensor that we refer to as the 5′ tethering pathway [5] can increase the affinity and/or Vmax of RNase E towards its substrate by one to two orders of magnitude. As a result, a 5′ monophosphorylated substrate is generally turned over more efficiently than the corresponding triphosphorylated form [81–83].

RNase E is a tetramer and the four subunits are arranged as a dimer of dimers in the crystal [63, 80]. This quaternary structure is likely to be a conserved feature, since RNase E orthologues from plants and mycobacteria have also been shown to form tetramers [84, 85]. The oligomerization of RNase E is important for catalytic activity. A substrate with a free 5′ P end can bind to the 5′ sensor of one protomer and be cleaved in the active site of the other protomer [86] (Fig. 1b). This model explains both the preference for 5′ P RNAs and why dimers or higher order complexes are required to express this preference [82, 86]. In principle, the length of a substrate RNA can be quite variable as long as the 5′ end and the site of cleavage are in a confirmation compatible with enzyme geometry. The architecture of RNase E has been reviewed extensively [87, 88].

**Fig. 3:**
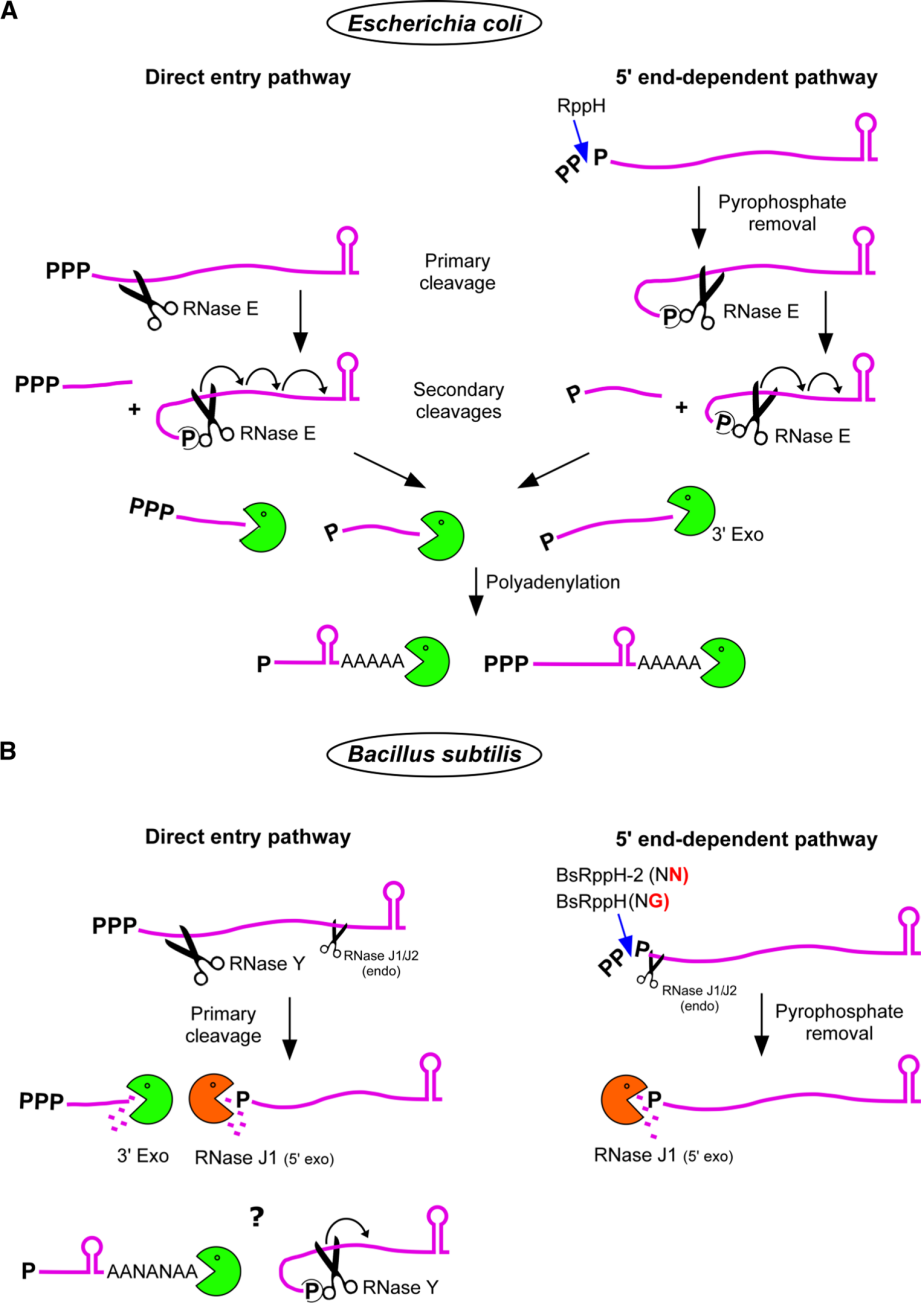
RNA degradation pathways in *E. coli* and *B. subtilis.* Initiation of mRNA decay as defined by the first nucleolytic cleavage can depend on a variety of parameters that render a given mRNA susceptible to the action of an RNase (e.g., translation efficiency, 5′ end conversion, stochastic events, etc., see text). **a** In *E. coli*, the major direct entry pathway involves a primary cleavage of the native transcript by RNase E. The upstream fragments are rapidly degraded by 3′ exoribonucleases (RNase II, PNPase, RNase R, and oligoribonuclease for short oligonucleotides). The 5′ monophosphorylated downstream fragment is preferentially recognized by the 5′ sensor of RNase E, which enhances the rate of subsequent cleavages (>20-fold, at least in vitro). This causes a wave of secondary downstream cleavages proceeding in a 5′–3′ direction each generating a 3′-OH upstream fragment that is degraded by 3′ exonucleases. Decay intermediates whether or not protected by 3′ secondary structure can be polyadenylated by poly(A) polymerase, enabling the 3′ exonucleases to re-engage several times if necessary to produce complete degradation (see main text). Polyadenylation can also be observed on full-length transcripts containing the transcription terminator (not shown in the figure) but does not represent a major pathway to initiate mRNA decay (see main text, “The 3′ end: tailing, scavenging and surveillance”). A second pathway of mRNA degradation in *E. coli* is 5′ end-dependent and starts with pyrophosphate removal by the pyrophosphohydrolase RppH. This tethers RNase E to the 5′ end of the transcript and stimulates downstream cleavage in the same way as described for secondary cleavages above. Refer to the legend of Fig. 1 and text. **b** In *B. subtilis*, the pathways initiating mRNA decay are similar to *E. coli* but the players are different. In the major direct entry pathway, the primary cleavage is affected by RNase Y and to a lesser extent by RNase J1/J2 or another endonuclease. The upstream fragments are degraded mainly by PNPase, in contrast to RNase II in *E. coli* [313–315]. The monophosphorylated downstream cleavage products are degraded 5′–3′ by RNase J1/J2 in exonuclease mode and can proceed to the 3′ end. It is interesting to note that the *B. subtilis* extracts used to demonstrate the largely phosphorolytic degradation of RNA to mononucleotides [313] most likely did not measure the contribution of the, at the time, unknown hydrolytic RNases J1/J2 to exonucleolytic decay, due to the 5′ triphosphorylated RNA substrate used and the fact that most of the ribosome associated RNase J was probably eliminated during extract preparation [313]. *B. subtilis* has no poly(A) polymerase but A-rich polynucleotide tails synthesized by an unknown enzyme (indicated by an ANA sequence) are found essentially on degradation intermediates [138]. The* question mark* indicates that it is not clear whether they contribute to the degradation of 3′ structured fragments. However, 3′ terminal fragments containing the transcription terminator are very resistant to 3′ exonuclease attack. The 5′ exonuclease activity of RNase J is thus very useful to degrade 3′ structured RNA fragments. Similar to *E. coli*, conversion of the native 5′ PPP to a 5′ P by BsRppH (which prefers a G in second position, see text) and BsRppH-2 (not yet identified, but insensitive to N-terminal sequence, see text) renders the mRNA susceptible to the 5′ exonuclease activity of RNase J. In vitro, RNase J can also cleave endonucleolytically a native transcript close to the 5′ end probably by threading the 5′ PPP through the RNA entry channel and past the 5′ P binding pocket. It is not known whether this “sliding endonuclease” mode plays a significant role in 5′ end conversion in vivo. Similarly, RNase Y activity is stimulated by a 5′ P group in much the same way as RNase E, but to what extent RNase Y competes with RNase J for binding to a monophosphorylated 5′ end in vivo remains to be analyzed (indicated by a *question mark*)

Before RNase E can enter the 5′ tethering pathway the original 5′ terminal triphosphate of an mRNA must be converted to a monophosphate (Fig. 3a). This conversion is catalyzed by the pyrophosphohydrolase RppH that preferentially acts on single-stranded 5′ termini [89, 90]. Interestingly, RppH, which belongs to the Nudix hydrolase family, is evolutionarily related to the eukaryotic decapping enzyme DCP2 which catalyzes a very similar reaction [91]. Since both RNase E and RppH rely on single-stranded 5′ termini to access their substrate, this explains the stabilizing effect of 5′ secondary structures that has been known for a long time [92–95]. On mRNAs known to decay primarily in a 5′ end-dependent manner (e.g., *E. coli rpsT*), mutating the RNase E 5′ sensor (Arg169Glu) causes a similar increase in stability as the absence of a functional RppH [96]. However, inactivation of RppH affects the stability of only about 10 % of all mRNAs in *E. coli* [90], suggesting that the decay of a majority of transcripts is initiated via other routes, notably the direct entry pathway (see below).

Interestingly, autoregulation of RNase E expression involves a primary cleavage within the *rne* UTR that is not sensitive to the presence of RppH (see below) but the autoregulation is abolished in a 5′ sensor mutant. This suggests that secondary cleavages that degrade the downstream *rne* open reading frame require stimulation by the 5′-P terminus produced by the initial cleavage [96]. This is one of the rare examples that documents the importance of a 5′ monophosphorylated RNA for RNase E activity in vivo. Pyrophosphate removal by RppH not only tethers RNase E to the 5′ end but also makes it more likely that the 5′ UTR rather than another segment of the mRNA will subsequently be cut, providing it contains suitable cleavage sites [97]. An RBS located in the leader region of a pathogenic *E. coli* mRNA has recently been shown to provide extensive protection against RNase E-mediated decay of the downstream (translated) mRNA [98]. However, it is unlikely that this strong protective effect would remain when translation of the mRNA is inhibited. Studies on the only known CsrA-mediated activation mechanism in *E. coli* provided another example of the important role of the 5′ end for RNase E-dependent decay. As part of a global regulatory system CsrA normally represses translation of numerous genes often leading to rapid mRNA decay [99]. However, CsrA activates *flhDC* expression, encoding the master regulator of flagellum biosynthesis and chemotaxis, by binding to two 5′ proximal binding sites in the *flhDC* leader and blocking the 5′ end-dependent RNase E cleavage pathway [100].

### Importance of the 5′ end for RNase J

In *B. subtilis*, the 5′ region of a transcript was recognized early on as a major stability determinant [101–105]. The presence at or near the 5′ end of a hairpin structure, a ribosome binding site or a bound protein can stabilize long downstream regions of an mRNA [106–111]. This protection, at a distance, is much more impressive in *Bacilli* than it is in *E. coli*, notably when the mRNA is not translated [5].

In *B. subtilis,* certain native transcripts when converted to 5′ monophosphorylated mRNAs become vulnerable to attack from the 5′ end (Fig. 3b), in much the same way as in *E. coli.* The *Bacillus* pyrophosphohydrolase also prefers single-stranded 5′ ends [112]. Mechanistically, BsRppH removes the γ and β phosphates as orthophosphate [112, 113], whereas EcRppH releases them primarily as pyrophosphate [90]. Purified BsRppH requires at least two unpaired nucleotides at the 5′ end but prefers three or more. In addition, a critical recognition determinant for the enzyme in vitro and in vivo is a G residue in the second position [114]. This preference is corroborated by 3D structural data of the *B. subtilis* pyrophosphohydrolase [115]. Analysis of 600 *B. subtilis* primary transcripts whose start points have been identified at single-nucleotide resolution [116] suggests a counter selection for guanosine residues in position 2 among primary transcripts [115]. However, *B. subtilis* mutants that lack RppH retain about 30 % of the RNA pyrophosphohydrolase activity of wild-type cell extracts [114]. The pyrophosphohydrolase responsible for this activity is unknown but in contrast to BsRppH it is sequence-independent [114].

Inactivation of *rppH* in *B. subtilis* has been shown to stabilize the *yhxA*-*glpP* transcript, which thus decays primarily via a 5′ end-dependent pathway. Maintaining the 5′ triphosphorylated end of the original mRNA was sufficient to protect it against the 5′ exonucleolytic activity of RNase J1/J2 [112]. The first step in this pathway (5′ end conversion) is equivalent in *E. coli* and *B. subtilis* but the subsequent steps differ significantly. The 5′ P RNA is destroyed via the 5′ tethering mechanism described above involving RNase E in *E. coli*, whereas in *B. subtilis* the mRNA is subject to the monophosphate-dependent 5′ exonuclease activity of RNase J1 (Fig. 3b). A rationale for the dependence of RNase J on a 5′ P in exonuclease mode has been obtained from the crystal structure of *Thermus thermophilus* RNase J in complex with UMP [58]. The 5′ monophosphate is coordinated by several serine and histidine residues that are part of a monophosphate binding pocket located just a single-nucleotide distance from the catalytic center (Fig. 2b). While 5′ P docking on RNase E does not give an immediate clue as to its potential role in modulating enzyme activity the interpretation is more straightforward in the case of RNase J. The one nucleotide distance between the 5′-P binding pocket and the active site immediately explains the preference of the enzyme for a monophosphate in exonuclease mode [58]. A 5′ terminal di- or trinucleotide sliding into the pocket would place the scissile phosphodiester bond out of phase with the catalytic center (Fig. 2c). However, the enzyme is able to initiate exonucleolytic decay of an RNA with a 5′ OH moiety as illustrated by the RNase J1-dependent degradation of the *glmS* mRNA following ribozyme induced self-cleavage [117].

A possible alternative model to render an RNA vulnerable to exonuclease attack from the 5′ end is based on the capacity of both *B. subtilis* RNase J1 and RNase J from *Mycobacterium* to endonucleolytically cleave very close to the 5′ end, at least in vitro [73]. In this mechanism, the native 5′ PPP RNA enters the RNA entry channel of the native dimer that continues past the active site (Fig. 2d) [118, 119] and is threaded towards the catalytic center in the same way as a 5′ P RNA. Since the 5′ PPP moiety cannot dock productively with the mononucleotide binding pocket it could slide past the active site and be cleaved endonucleolytically at any of the first few nucleotides. The cleavable phosphodiester fits into the monophosphate binding pocket much in the same way as the 5′ terminal monophosphate [118] so the following phosphodiester groups should be readily cleaved endonucleolytically once the 5′ PPP group has slid past the active site [73]. In addition, this “sliding endonuclease” mode would also be expected to be very sensitive to secondary structure and is thus not likely to be used for cleavage of sites further in the body of a mRNA. Whether this sliding endonuclease activity of RNase J plays a significant role in vivo is unknown.

### Importance of the 5′ end for RNase Y

Another route to initiate mRNA degradation in *B. subtilis* involves RNase Y. This enzyme has initially been characterized as an endoribonuclease which, like RNase E, prefers a monophosphorylated RNA as a substrate, at least in vitro [59]. As such, RNase Y could compete with RNase J for binding to the 5′ terminal phosphate (Fig. 3b). However, for one template, it has been shown that following RppH-dependent 5′ P conversion of the *yhxA*-*glpP* mRNA, RNase Y does not contribute significantly to the 5′ P-dependent decay, which only depends on the 5′ exonucleolytic activity of RNase J1 [112]. Nevertheless, in this case one would not necessarily expect RNase Y to cleave this particular transcript internally since it was selected for being degraded primarily via the 5′ end-dependent pathway. More generally, from a few mRNAs studied to date it appears that RNase Y can cleave mRNA efficiently in vivo without a requirement to tether to a 5′ P (see below). The activity of RNase Y in vitro is also very sensitive to secondary structure. This sensitivity does not only reflect the requirement for single-strandedness of the region to be cleaved but maybe also exhibit a certain preference for secondary structure 3′ to the cleavage site [59]. Substrate recognition by RNase Y might thus be quite complex and clearly requires further analysis using different RNAs.

## The 3′ end: tailing, scavenging, and surveillance

Transcription of bacterial mRNAs usually ends at an intrinsic transcription terminator. This secondary structure protects the mRNA 3′ end from exonucleolytic attack. In *E. coli,* the original mRNA or decay intermediates generated by endonucleolytic cleavage, whether or not protected by 3′ secondary structure, can be polyadenylated by poly(A) polymerase (PAP I). This enables the 3′ exonucleases to re-engage several times if necessary to produce complete degradation (for recent reviews, see [120, 121]). In some cases, 3′ polyadenylation can indirectly control the functional mRNA level [122]. Poly(A) polymerase activity is stimulated by 5′ phosphorylation of the RNA (as generated by endonucleolytic cleavage) and by the RNA chaperone Hfq [123–126]. Although most mRNAs in exponentially growing *E. coli* cells are polyadenylated to some extent [127], only ~2 % of total RNA is polyadenylated at any given time [125]. It has been proposed that the slow rate of addition of the first A-residues (0.5–7 nts/min) combined with the fast removal of longer poly(A) tails by the 3′ exoribonuclease II explains why full-length transcripts are primarily degraded by the major RNase E-dependent pathway [128].

Deletion of the *pcnB* gene encoding poly(A) polymerase has only a minimal effect on growth rate [129]. However, deregulation of PAP I is associated with slow growth or lethality [130, 131], and interestingly, this effect is not related to RNA quality control but rather to a direct role in depleting functional tRNA levels [132].

The current consensus is that polyadenylation acts, at least in *E. coli*, as a scavenging and surveillance mechanism whose primary function is to accelerate the decay of 3′ structured degradation intermediates and to get rid of mRNAs that accumulate abnormally when the principal decay pathway is not operational [120, 133, 121]. Poly(A) polymerase is not the only enzyme capable of tailing 3′ ends; in its absence, long (>30 nt) A-rich polynucleotide tails can still be observed in *E. coli*. PNPase has long been known to be a reversible enzyme that can either degrade RNA by using inorganic phosphate or synthesize RNA by using NDPs as precursors [134, 135]. Due to the high intracellular levels of inorganic phosphate (>10 mM) [136] it was thought that this enzyme works exclusively as an exoribonuclease in vivo, a hypothesis proved wrong by the discovery that PNPase is the second enzyme in *E. coli* responsible for the non-templated addition of A-rich polynucleotide tails to the 3′ ends of RNA [137].

Long heterogenous tails have also been characterized in *B. subtilis* [138],* Streptomyces* [139], and Cyanobacteria [140], but not in Mycobacteria [141]. No true *E. coli* PAP homologue has been identified in these species but a PNPase orthologue has been implicated in generating polynucleotide tails in *Streptomyces* [142, 139]. In *B. subtilis,* the polyadenylation profile comprising both short poly(A) and polynucleotide tails with a mean size of 40 nt remains almost unchanged in the absence of PNPase [138], and no polymerase responsible for the 3′ tailing has yet been identified. Moreover, tailing was almost exclusively detected on degradation intermediates which might be a bias of the method used [138] but which fits well with the observation that RNAs with structured 3′ ends are very resistant to 3′ exonucleolytic attack in *B. subtilis.* Poly(A) assisted degradation of structured 3′ ends would also be much less important than in *E. coli* since *B. subtilis* RNase J1 can efficiently degrade RNA fragments containing the transcription terminator from the 5′ side following an initial endonucleolytic cleavage [59, 143].

At present, there is no evidence that long heterogeneous tails affect RNA stability in bacteria. In *E. coli,* the addition of five A-residues to an RNA 3′ end incorporated into a stable stem-loop structure is sufficient to stimulate exonucleolytic degradation [144], suggesting that longer hetero- or homopolymeric tails may have a different function in RNA metabolism.

## The direct entry pathway

### *E. coli* RNase E in direct entry mode

Bulk mRNA stability and the abundance of a majority of transcripts in *E. coli* appear to be much less affected by disruption of RppH than RNase E [90], but even before the discovery of RppH there was speculation that RNase E might initiate mRNA decay without being tethered to the 5′ end, in a pathway called the “internal entry” or “direct entry” model [5, 11, 60, 145, 146]. The precise mechanism of this pathway is not understood but appears to require the C-terminal half of RNase E (CTH). This region of RNase E is not essential for catalytic activity and its removal has only a moderate effect on bulk mRNA stability [11, 147]. However, the CTH is important for the rapid breakdown of many untranslated mRNAs [148] and the autoregulation of *rne* expression, as the *rne* mRNA is stabilized in a ∆CTH RNase E mutant [149]. It might also selectively affect the abundance of transcripts involved in certain metabolic pathways [10]. In addition, the CTH is also required to recruit RNase E to mRNAs that are translationally repressed by sRNA [150, 151]. No individual domains (e.g., RNA binding sites) of the CTH have so far been identified that significantly affect the direct entry pathway. Similarly, none of the multiple RNA binding proteins that interact with the CTH to form the degradosome (e.g., PNPase or RhlB) has been specifically implicated in the direct entry pathway [150, 151].

Significantly, inactivation of the 5′ end-dependent pathway, either by inactivating RppH or mutating the phosphate binding pocket of RNase E, is synthetically lethal when combined with a ∆CTH RNase E mutation [47, 96]. This can be seen as genetic evidence that one of the two pathways initiating mRNA decay in *E. coli* has to remain functional. However, in vitro and crystallographic data also support a model where the direct entry pathway can occur with only the catalytic N-terminal half of RNase E. Internal flexibility observed within the quaternary structure of *E. coli* RNase E could account for the recognition of structured RNA substrates in the absence of 5′ end recognition [80]. In addition, based on kinetic studies Kime et al. [152] identified the minimum substrate requirement for 5′ end-independent cleavage of different RNAs. It appears to consist of multiple single-stranded segments in a conformational context that allows their simultaneous interaction with RNase E. Since single-stranded segments are frequently encountered in an mRNA, this model offers a simple explanation for the susceptibility of untranslated transcripts to RNase E [5]. Moreover, these segments could work cooperatively with a 5′ monophosphate when available and stretch over a considerable sequence length, which would allow contacts with the protomers of the principal RNase E dimer (Fig. 1b). The model proposed by Kime et al. [151] is also attractive in the sense that single-stranded regions could be bound with high affinity without being cleaved which would lower the entropic barrier and enhance the rate of cleavage at bona fide cleavage sites. Interestingly, RNase E can probably also recognize single-stranded sequences contained within a stem-loop structure, similar to the binding to the hairpin in the 5′ UTR of the *rne* transcript that is required for autoregulation of RNase E in *E. coli* [153]. Assuming that these in vitro observations are also relevant in vivo, there are thus multiple permutations of mechanisms that co-exist in *E. coli* to initiate mRNA decay by the direct entry pathway. It can also be mentioned that a CTH-independent mechanism could notably be used in bacteria, which only contain short RNase E/G-like enzymes equivalent to the N-terminal half of *E. coli* RNase E, as encountered in many *Bacilli* and *Clostridia* for example. However, at present, it is unknown how these short RNase E/G type enzymes present in many species contribute to mRNA metabolism.

### Direct entry in bacteria with RNase Y orthologues

As we have seen above there are many organisms that do not have an RNase E/G type enzyme (Table 1). Instead, these bacteria have an RNase Y orthologue, often together with RNase J, but some organisms only have one or the other. In *B. subtilis,* RNase Y is the only known ribonuclease capable of affecting bulk mRNA stability to a degree approaching that of RNase E in *E. coli*. This observation demonstrated that endonucleolytic cleavage plays a major role in mRNA metabolism in a Gram-positive organism lacking RNase E [59]. Transcriptome analyses of RNase Y-depleted strains have confirmed a predominant role of RNase Y in initiating not only mRNA but also non coding RNA decay/processing [49, 154, 155]. These studies reveal a cumulative non redundant total of about 1,600 mRNAs and several hundred non coding RNAs that are upregulated following RNase Y depletion but the individual studies differ significantly in the identity of the RNase Y targets [154]. This indicates that the experimental conditions are extremely important for the outcome of the experiment. Differences in medium, growth conditions, degree of RNase depletion and statistical data evaluation are all critical. A single experimental condition does not permit identification of all or even a majority of the major RNase Y substrates. The role of RNase Y in RNA degradation and gene regulation has been studied in more detail for a handful of transcripts. They include the *gapA* operon [62], S-adenosylmethionine (SAM) riboswitches [59], *rpsO* [143], the *infC*-*rpmI*-*rplT* operon [156], *mreBH*-*ykpC* and *spoIISAB* [50], the *bsrG*/SR4 type I toxin-antitoxin system [157], the *tag* regulon and *dnaA* [154]. A number of cleavage sites have been mapped precisely and they are shown in Table 2. Common features include single-strandedness, an enrichment in AU residues and a proximity to secondary structure. These characteristics are reminiscent of known *E. coli* RNase E cleavage sites. Cleavage at site 1 in the *B. subtilis yitJ* leader (Table 2) has been reproduced with purified RNase Y using riboswitch RNA bound to SAM, the same configuration in which cleavage most likely occurs in vivo. In vitro, RNase Y cleaves this substrate significantly faster in its 5′ monophosphorylated form compared to the 5′ triphosphorylated RNA [59]. However, in vivo the upstream cleavage product containing the 5′ end accumulates to very high levels in the absence of 3′ exonucleases, suggesting that no significant conversion of the original 5′ PPP terminus to 5′ P that would allow exonucleolytical degradation by RNase J1/J2 from the 5′ end, occurs. Therefore, in vivo RNase Y cleaves the SAM riboswitch most likely via a direct entry pathway, i.e., without tethering to the 5′ terminus [59]. Similarly, available data suggest that 5′ tethering is likely not required for the cleavage of other known RNase Y substrates, the *rpsO* [143], *ermC* [158] and *infC* transcripts [156]. This does not exclude that RNase Y, again like RNase E, could cleave other substrates also more efficiently in vivo when a 5′ P terminus were available. Settling this point probably require the identification or construction of a suitable substrate. For example, a known RNase Y cleavage site could be integrated into an mRNA decaying primarily via the 5′ end (RppH)-dependent pathway. In an RNase J1/J2 deletion mutant, which lacks both the 5′ exonuclease and the endonuclease activity of RNase J, internal cleavage efficiency by RNase Y could then be measured as a function of RppH activity.

**Table 2:** Known endonucleolytic cleavage sites for RNase Y and J

RNase Y		
Bs *yitJ* C1*	(--)GACACGAAAAUUU^CAUAUCCG(--)	[59]
Bs *yitJ* C2	(--)GAGACA^AAAUCACUGAC(--)	[59]
Bs *gapA*	(--)CAAAGAA^GU(--)	[62]
Bs *infC* C1	(--)TATTG^TGTAGAATAGT	[156]
Bs *infC* C2	(--)TGACCGTAC^ATTTTTATTGA	[156, 317]
Sa *sae*	TATACAACTAT^TAAATCCCATAA	[3]
RNases J1 and J2		
Bs *thrS* leader**	GAUUCCG^UUUAUUC	[16, 161]
Bs *thrZ* leader	CCACGGG^UUAAUCA	[16, 161]
Bs *trp* leader	CAUUAUG^U^U^UAUUC	[318]
Bs *ilv*-*leu*	GAGAACA^GGUACA	[1, 163]
Bs scRNA	AUCAUCA^AAUUUUC	[162]

The ^ symbol marks the site of cleavage. Dashes in parentheses indicate a secondary structure. The * asterisk* indicates the only RNase Y cleavage site demonstrated to occur in vivo and in vitro. Two* asterisks* show that this cleavage site can be cleaved by RNases J, Y, and E [24]

*Bs* *Bacillus subtilis*, *Sa* *Staphylococcus aureus*, *C* cleavage site, *scRNA* small cytoplasmic RNA

In *S. aureus*, an RNase Y-like protein, CvfA, was initially identified as a novel virulence regulator that hydrolyzes the phosphodiester linkage in cyclic nucleotides [159]. However, CvfA clearly is a functional homolog of RNase Y that seems to have a more restricted effect on global gene expression than its *B. subtilis* counterpart. Interestingly, among a total of 569 transcripts with altered abundance in a CvfA/RNase Y mutant strain of *S. aureus* half corresponded to intergenic regions and non coding RNAs [3]. The CvfA/RNase Y processing site identified in the primary *saePQRS* mRNA (encoding a global virulence regulator system) resembles those described for *B. subtilis* (Table 2). Similarly, in another Gram-positive pathogen, *Streptococcus pyogenes,* inactivation of the RNase Y ortholog CvfA can alter the expression of up to 30 % of the transcriptome in stationary phase, including multiple virulence genes [78]. However, the steady-state levels of most mRNAs are not significantly affected during exponential growth despite a twofold increase in bulk mRNA stability [20, 78]. These contradicting observations might be explained by an altered mRNA synthesis rate in the *cvfA/rny* mutant but this hypothesis remains to be verified [20]. At present, it is unknown whether the 5′ sensitivity observed in vitro with *B. subtilis* RNase Y is conserved in orthologous enzymes in other species and whether it plays a role in vivo.

### The potential role of RNase J1/J2 in direct entry

RNases J1 and J2 were originally identified as endoribonucleases that can cleave the *thrS* 5′ UTR in vitro upstream of a leader terminator structure. This site was cleaved with equal efficiency in 5′ mono- and triphosphorylated transcripts indicating that endonucleolytic cleavage by RNases J1 and J2 is not sensitive to the nature of the 5′ end [16]. Cleavage of a second upstream site in the *thrS* leader that was only observed on the 5′ P substrate ([16], and much less efficiently with RNase J2 compared to RNase J1) is, as we know now, not endonucleolytic but the result of a block to 5′ exonuclease progression [58]. Consistently, RNase J2 has recently been shown to be an inefficient 5′ exonuclease [160].

Further evidence that RNases J1/J2 have endonucleolytic activity in vivo was obtained by studies on the *thrZ* mRNA, encoding a second threonyl-tRNA synthetase. The original *thrZ* transcript is efficiently processed upstream of a leader terminator, a configuration very similar to that found in the *thrS* leader [161]. The 5′ end of the primary *thrZ* transcript that is located 800 nts upstream of the processing site is only detectable in a RNase J1/J2 double mutant [16] but RNase Y may also be involved in 5′ UTR cleavage (see below). As an endoribonuclease, RNase J1 is also implicated in the maturation of the small cytoplasmic (sc)RNA [162], the processing of the *ilv*-*leu* polycistronic transcript [163] and in the turnover of the *trp* leader RNA [164].

RNases J1 and J2 initially isolated from a ribosomal high salt wash co-purified in stoichiometric quantities despite a different individual chromatographic behavior suggesting that they exist as a hetero-oligomeric complex in vivo [16]. The existence of the RNase J1/J2 complex that likely is a heterodimer under physiological conditions has been confirmed and, interestingly, the mixed complex has a somewhat different endonucleolytic cleavage specificity in vitro as compared to the individual enzymes [160].

Transcriptome and proteome studies of RNase J1/J2 mutants clearly point to an important general role of these enzymes in RNA metabolism, with hundreds of transcripts being affected [163, 50]. However, a reliable assessment of the importance of RNase J endonuclease activity is complicated for two major reasons. First, the dual activity of RNase J is based on a single catalytic center and mutations generally affect both activities. In addition, the proximity of the monophosphate binding pocket to the catalytic center suggests that once cleaved endonucleolytically, RNase J can probably switch to exonuclease mode on the same substrate [58]. Second, RNase Y has a cleavage specificity very similar to that of RNase J1/J2 [59] and, for example, the *thrS* leader can actually be cleaved in vitro by both enzymes at the same position [24]. As described above, RNases J1/J2 process the *thrZ* leader RNA in vivo. A recent tiling array study of an RNase Y depleted strain now suggests that RNase Y can also cleave the 5′ UTR of the *thrZ* mRNA [154]. Thus, some overlap in substrate recognition most likely also occurs in vivo.

In group A *Streptococci* (GAS), two categories of mRNAs have been described. Class I transcripts are unstable in both exponential and stationary phase, whereas class II mRNAs that code for a number of virulence related proteins are resistant to nucleolytic attack for up to 20 min in stationary phase before being degraded. It has been proposed that RNases J1 and J2 initiate decay through endonucleolytic cleavage [77, 165]. In their model, the authors suggest that class I transcripts are efficient substrates that titrate RNase J before becoming available to cleave class II mRNAs.

There are arguments that can be invoked to explain why the endonucleolytic activity of RNase J might be less relevant than that of RNase Y in vivo. Recent crystallographic data on RNase J bound to an RNA suggest that in order to directly accommodate a substrate in endonucleolytic mode, without threading the RNA through the RNA entry channel as in exonuclease mode, the two subunits of the dimer must separate or at least “breathe” [118, 119]. In addition, high enzyme concentrations are generally required to observe cleavage by RNase J1/J2 in vitro. However, it should be noted that RNase Y activity in vitro requires similar enzyme concentrations as that of RNase J1/J2 [59]. Poor in vitro cleavage is thus not a good indicator for lack of physiological relevance. The few substrates tested so far might simply not be presented to the enzyme in the optimal conformation and/or the enzyme itself requires a co-factor and/or different context for efficient cleavage to occur. This co-factor might be a component of the ribosome to which RNase J is most likely localized [16, 166] and which could help to accommodate an RNA in endonucleolytic mode. Probably the most solid evidence that RNase Y out-competes RNase J as an endonuclease in vivo is its significantly stronger effect on bulk mRNA stability [16, 59].

## The role of translation

Translating ribosomes are one of the most important factors influencing the lifetime of a bacterial mRNA. Impaired translation often accelerates mRNA decay. This relationship provides a quality-control mechanism that minimizes the production of abnormal and potentially harmful proteins from poorly or improperly translated mRNAs. We will focus here on more recent advances and refer the interested reader to earlier reviews of this topic [5, 167].

There exists a variety of ways by which the presence of ribosomes can protect a transcript from initial attack by ribonucleases. In addition, the effect of translation on mRNA decay also depends on the nature of the ribonucleases present in a given organism.

### Effect on RNase E cleavage

In *E. coli*, active translation often protects the mRNA against an attack by RNase E. This implies that ribosomes are required for directly shielding one or more cleavage sites within or close to the open reading frame. A good example is the *rpsO* mRNA that contains a major RNase E site only ten nucleotides downstream of the stop codon. Terminating translation artificially 20 nucleotides further upstream is sufficient to significantly increase cleavage and destabilize the mRNA [168]. RNase E can thus relay translation efficiency of an mRNA to chemical decay. Uncoupling transcription and translation is another way to study the protective effect of translating ribosomes. When the *lacZ* mRNA is transcribed by T7 RNA polymerase, which is resistant to polarity [169], long stretches of the mRNA are “naked” because the ribosomes cannot keep pace with T7 polymerase that transcribes several fold faster than the *E. coli* enzyme. These ribosome-free regions are prone to RNase E attack, and the transcript becomes even more unstable when translation is abolished altogether [146]. On the other hand, mRNA cleavage by the MazF toxin in *E. coli* (see below) can be enhanced when the mRNA is actively translated, probably by removing secondary structure [170]. MazF being much smaller than the RNase E degradosome complex can likely access its cleavage sites between translating ribosomes more efficiently.

However, also in *E. coli* there are also a number of cases where large fragments of mRNA can remain untranslated without being excessively unstable [169, 171, 172]. For instance, translation of about one-fifth of the *bla* mRNA is sufficient to stabilize the remaining 80 % of the mRNA that would otherwise be labile [172]. A similar effect is observed in the case of the *puf* operon in *Rhodobacter capsulatus,* whose decay is controlled by an enzyme closely related to *E. coli* RNase E [173]. The mRNA encoding the two promoter-distal cistrons *pufL* and *pufM* is stabilized as long as ribosomes are present over the first two promoter-proximal cistrons *pufAB* and the beginning of *pufL,* but not over the major cleavage site located downstream [171].

Thus, direct shielding of RNase E cleavage sites is not always required and ribosomes can provide protection “at a distance”. This intriguing difference has been proposed to reflect the way RNase E interacts with its target transcripts, mainly the 5′ tethering pathway (protection “at a distance”) and direct entry (shielding by translating ribosomes) [5]. In accordance with this view, the *bla* mRNA can be stabilized by appending a 5′ hairpin [95], which is known to impede the 5′ end conversion by RppH [90]. This mRNA is thus likely to follow the 5′ tethering pathway. In contrast, the stability of the *rpsO* mRNA and the *lacZ* mRNA transcribed by T7 RNA polymerase are not affected by structurally sequestering the 5′ end [146, 174] and these transcript are therefore likely to be degraded by the direct entry pathway.

In both pathways, the ribosome binding site and the 5′ UTR play an important role. A strong RBS directs efficient translation initiation allowing closer spacing of translating ribosomes and potentially improved steric protection. A number of studies clearly indicate that efficient ribosome binding to the RBS helps to protect mRNAs from ribonuclease attack [167]. At the same time, the RBS region, which often is relatively unstructured [175], as well as the ribosome-free 5′ UTR could a priori constitute a preferred region for cleavage by RNase E [5]. This was confirmed in a recent study that analyzed the influence of translation on the 5′ tethering and direct entry pathway, respectively. Indeed, poor ribosome binding favors degradation by both pathways but the effect on the 5′ end-dependent decay is stronger [97]. This suggests that RNase E, after engaging a monophosphorylated 5′ terminus, searches nearby for a cleavage site preferring those that do not require the enzyme to reach around intervening ribosomes [5, 97]. Accordingly, cleavages in the ribosome-free 5′ UTR are favored, provided a suitable cleavage site is present there. This behavior contributes to the overall 5′–3′ direction of RNase E-mediated mRNA decay as defined by an orderly wave of successive cleavages. This pathway might not always be valid but nevertheless is the biologically most efficient decay mechanism [6, 176].

The fact that large segments of mRNA can remain unprotected by ribosomes (e.g., the *bla* mRNA) without being excessively unstable suggest that bona fide RNase E target sites are rare within coding sequences [171, 172]. This implies that genuine cleavage sites which are intrinsically vulnerable to attack by RNase E should be of a different nature compared to the secondary cleavage sites that are only recognized in the context of a wave of 5′–3′ decay (Fig. 3a) [6]. Therefore, does binding to the 5′ end of an RNA alter the cleavage specificity of RNase E, i.e., can low affinity sites become cleaved more rapidly? To our knowledge, this intriguing question has not been addressed experimentally.

### In the absence of RNase E

A quite different picture of the interplay translation-mRNA decay emerges when we look at organisms that do not contain RNase E like many Gram-positive *Bacilli.* From early on, it became apparent that translation of the body of an mRNA might not be a major determinant of transcript stability [101, 104]. Instead, the 5′ end and the translation initiation region appear to have a key role in protecting an mRNA against nuclease attack. Several 5′ leader regions from long-lived mRNAs (e.g., *ermC, atpE, cryIIIA*) are capable of strongly stabilizing the entire open reading frame in the absence of translation [106, 109, 110, 177]. Steric occlusion of the 5′ end and/or a strong Shine-Dalgarno sequence, even without an associated translation initiation codon are the common determinants to observe this effect. A variety of sequences can be stabilized when fused to these stability-conferring leader regions, including very long untranslated transcripts such as the *E. coli* *lacZ* mRNA [109, 178, 179]. This illustrates that protection at a distance is much more efficient in *Bacilli* than in *E. coli.* Indeed, in the latter a stably bound ribosome at the 5′ end cannot protect the downstream *lacZ* mRNA against RNase E [146]. Assuming that the endonuclease activity of RNase J1/J2 is not very significant under physiological conditions (which remains to be shown) the 5′ exonuclease activity of RNase J1 could perfectly explain the enormous potential of 5′ stabilizing elements in *Bacilli.* However, how does the globally acting RNase Y which has an in vivo and in vitro cleavage specificity similar to RNase E [59] fit into this scenario? First of all, the enormous stability of 5′ protected but untranslated *E. coli lacZ* mRNA observed in *B. subtilis* [109, 179] clearly suggests that RNase Y cannot efficiently cleave this transcript internally, compared to RNase E when the same transcript is expressed in *E. coli.* This illustrates that *B. subtilis* RNase Y and *E. coli* RNase E may have similar but not identical cleavage specificity. It is possible that RNase Y is more demanding in the selection of cleavage sites than RNase E and that, as a consequence, the decay of a number of transcripts is simply not initiated by RNase Y cleavage. This would also explain why certain mRNAs (e.g., *epr, sacA, sacB*, and *penP)* can be efficiently stabilized in vivo when fused to a 5′ stabilizer (e.g., the *ermC* ribosome stall sequence, [110]). In agreement, the abundance of these transcripts is not significantly increased in a strain depleted for RNase Y [154].

On the other hand, the absence of specific cleavage sites in a handful of even very long mRNAs is, in our view, not synonymous with the notion that translation of an mRNA plays no role in determining its stability on a genomic scale in *B. subtilis*. For example, the decay of the *rpsO* mRNA is initiated by an RNase Y cleavage within the open reading frame [143], similar to the RNase E initiated decay of the orthologous mRNA in *E. coli* [168]. RNase Y cleavage of the *gapA* operon transcript also takes place within an open reading frame [62]. In the absence of more conclusive data, there is no obvious reason why translation might not affect RNase Y (or endonucleolytic RNase J1/J2) cleavage within an open reading frame. Global deep-sequencing approaches using RNase J1/J2 and RNase Y knock-out mutants should allow us to obtain a more complete picture of endonucleolytic cleavage/processing sites.

In *B. subtilis,* the predominant role of RNase Y in initiating mRNA decay is closely coupled with the 5′ exonuclease activity of RNase J1. For example, in a number of cases initial cleavage by RNase Y takes place within the 5′ UTR of a mRNA. The fate of the open reading frame then depends essentially on the efficiency with which RNase J1 destroys the downstream ribosome binding site through its exonuclease activity. In this case, the intracellular level of the mRNA open reading frame should depend on the activity of both RNase Y and RNase J1/J2, a scenario observed for the *tagD* mRNA coding an essential enzyme for cell wall biosynthesis [154]. Similarly, most of the *hbs* mRNA is found to be trimmed by RNase J1 to the translation initiating ribosome following an endonuclease cleavage by an unknown nuclease [180]. Cleavage by RNase Y near the 5′ end of the *infC*-*rpmI*-*rplT* polycistronic mRNA creates an entry site for RNase J1, which renders the stability of the *infC* mRNA dependent on the efficiency of translation initiation at the first cistron. In the absence of RNase Y cleavage, the 5′ proximal sequences specifically inhibit translation of *infC*, encoding the essential translation initiation factor IF3 [156]. By controlling at least partially the intracellular concentration of IF3, RNases J1 and Y can thus provide a link between RNA decay and translation.

If this decay scheme was valid on a large scale one could expect to observe a large number of transcripts upregulated by the depletion of either RNase Y or RNase J1/J2. A comparison of available transcriptome data shows a relatively low overlap, ranging from less than 10 % to about 25 % [50, 154, 163]. These numbers are probably a low estimate because only transcripts cleaved close to the 5′ end, which leave the open reading frame mostly intact, would register as RNase J1-dependent. Nevertheless, these results would be consistent with the notion that a significant number of transcripts could be cleaved endonucleolytically by RNase J1/J2 followed by 5′ exonuclease degradation.

In *S. aureus*, genome-wide antisense transcription has been associated with about 50 % of the genes [51, 52]. This pervasive low-level antisense transcription leads to the digestion of overlapping sense/antisense transcripts by RNase III and generates short (<50 nts) RNAs [51]. To what degree this process contributes to modulate the level of sense RNAs is unknown [181]. In comparison, similar antisense transcription in *B. subtilis* is much less extensive and only concerns about 13 % of the genes [182].

As already mentioned, translation does not always exert a protective effect against ribonuclease action but can actually also facilitate the endonucleolytic cleavage of an mRNA as in the case of the MazF toxin. However, cleavage of translated mRNAs can also be initiated in a number of situations that cause ribosome stalling [5]. Recently, the Aiba group showed that amino acid starvation causes internal cleavage of the mRNA at or near the “hungry” codons [183]. The experimental conditions did neither induce the RelE toxin nor was the effect dependent on ppGpp. In addition, mRNA cleavage was still observed in the absence of five characterized toxin-antitoxin systems in *E. coli* [183]. Since no identified nuclease is involved in this process, a straightforward explanation attributes this effect to the ribosome itself which thus turns into a “killer ribosome” [5]. However, stalled ribosomes do not cleave their mRNA in vitro, even in the presence of tmRNA [184]. At present, only HrpA, a putative RNA helicase has been invoked to contribute to ribosome-mediated mRNA cleavage but its precise role remains to be established [185].

## Multiprotein complexes

In many bacteria, key enzymes of RNA metabolism assemble to form degradosome-like complexes, which are thought to streamline degradation pathways by merging related activities into compact molecular machines. The paradigm for such multi-enzyme complexes is the *E. coli* RNase E-based degradosome (Fig. 1a) [186, 187]. The RNase E N-terminal half comprises the globular catalytic domain while the C-terminal half, which is predicted to be disordered, provides the scaffold for the assembly of the degradosome. Within this naturally unfolded region a number of small domains likely able to adopt stable secondary structures recruit the other degradosome components: the DEAD box helicase RhlB, enolase and PNPase (Fig. 1a). Since RhlB is present in the cell in roughly equimolar amounts to RNase E, and enolase and PNPase are present in large excess, it is likely that RNase E exists in the cell essentially in the form of the degradosome [188, 189]. However, alternative helicases (i.e., CsdA, SrmB, and RhlE) can be recruited into the degradosome in response to cold shock or in stationary phase, conditions that interfere with the biogenesis of the ribosome [190–193].

The presence of enolase in the degradosome suggests a link between carbon metabolism and mRNA decay. Approximately 5–10 % of enolase is sequestered in the *E. coli* degradosome [194] and its absence from the complex significantly increases the half-lives of many mRNAs that code for enzymes involved in energy-generating pathways [10]. In response to phosphosugar stress, the sRNA-mediated rapid degradation by RNase E of the *ptsG* mRNA encoding the glucose transporter depends on the presence of enolase [195].

Moreover, a large number of other proteins are found in sub-stoichiometric amounts on degradosomes purified from cell extracts. They include RNase R, polyA polymerase, Hfq [190, 196], protein chaperones GroEL and DnaK, ribosomal proteins [197–199], and polyphosphate kinase [200]. A more detailed description of the structural and functional aspects of the *E. coli* degradosome can be found elsewhere [30, 88]. RNA quality control and global post-transcriptional regulation are probably the major advantages afforded by RNA degradosome formation. How exactly this complex adds value to the degradation machinery clearly requires more investigations. For example, we still do not know what features the degradosome recognizes in an mRNA when selecting cleavage sites via the direct entry pathway, nor what are the contributions of the different components within the complex. Nevertheless, at least in *E. coli* the degradosome confers a clear selective advantage when wild-type cells are grown in competition with cells unable to form the degradosome [148].

RNase E-based degradosome assemblies of varying composition have been characterized in a number of proteo- and Actinobacteria but the interaction between enolase and RNase E might be restricted to enterobacteriales, pasteurellales, and vibrioales [88, 201]. However, RNase E in psychrotrophic γ-proteobacteria apparently does not associate with enolase, as is the case in *Pseudoalteromonas haloplanktis* [201] and *Pseudomonas syringae* [202]. In the latter, RNase R replaces PNPase in the complex which may be advantageous for degradosome-mediated decay of structured RNAs at low temperatures [203]. Other variants of RNase E-based degradosomes are found in the α-proteobacteria. In *Rhodobacter capsulatus,* RNase E forms a complex with two DEAD-box helicases and transcription factor Rho [204] and in *Caulobacter crescentus,* enolase is replaced by the Krebs cycle enzyme aconitase [205], a protein which has been shown, at least in *Mycobacterium tuberculosis*, to possess an iron-dependent RNA-binding activity [206].

Multi-enzyme degradative complexes of similar composition appear to exist also in organisms that lack RNase E. In *B. subtilis,* based on in vivo crosslinking and bacterial two-hybrid experiments, RNase Y has been proposed to organize a degradosome complex comprising enolase, phosphofructokinase, the RNA helicase CshA, PNPase and the endo-/exonuclease RNase J1/J2 [62, 207, 208]. However, unlike in *E. coli* [186, 194] or *C. crescentus* [205], the *B. subtilis* degradosome cannot be isolated in the absence of cross-linking agents. The direct interaction of RNase Y with enolase has been confirmed by native mobility-shift experiments [209] but the same authors found no evidence for an interaction between RNase J1 and RNase Y using a number of in vitro approaches. The recruitment of RNase J1/J2 into the RNase Y-based assembly thus remains subject to debate especially as this interaction could not be observed in yeast two-hybrid screens [160]. It is also difficult to reconcile the existence of a RNase Y-RNase J1 complex with the observation that RNase Y is bound to the membrane while the bulk of RNase J1 is most likely bound to ribosomes in vivo ([166], the *rnjA* gene is named *ykqC* in this publication) and that RNase J was initially purified from a ribosomal high-salt wash [16].

The RNase Y orthologue CvfA from *S. aureus* interacts with enolase in yeast two hybrid screens but this interaction has not yet been validated by direct purification techniques [78]. Based on bacterial two-hybrid screening, a degradosome complex in with a composition similar to that proposed for *B. subtilis* has been described in *S. aureus* [210]. In *Helicobacter pylori*, RNase J is associated with translating ribosomes and forms a complex with RhpA, the only DexD-box RNA helicase present in this organism. Complex formation stimulates the catalytic activity of both partners, i.e., the ATPase activity of RhpA and the capacity of RNase J to degrade double-strand RNA in vitro [211]. However, the RhpA helicase does not appear to interact with the *H. pylori* RNase Y orthologue [211].

Degradosomes based on protein–protein interactions are the rule. There are however other possibilities. Many bacteria contain an ortholog of the Ro autoantigen that binds a family of noncoding RNAs (ncRNAs) called Y RNAs [212]. In the extremophile *Deinococcus radiourans,* Y RNA can act as an adaptor between the Ro protein orthologue Rsr and PNPase and adapts the latter for effective degradation of structured RNAs. The small RNA physically docks the ring-shaped Rsr protein onto the exonuclease; Rsr then probably channels single-stranded RNA into the PNPase cavity [213]. This sRNA assembled degradation machine appears to be conserved in *Salmonella typhimurium* [213]. The ability of RNA to serve as a scaffold for molecular machines indicates another important parameter to understand interaction networks and opens new perspectives of how the substrate specificity of an enzyme can be modulated.

In conclusion, the compositional variation of the degradosome assemblies can be seen as a reflection of its capacity to optimize RNA decay/processing, by potentially integrating metabolic signals into this process and to adapting to environmental signals and optimizing growth in ecological niches.

## Cellular localization

Bacteria are not compartmentalized by internal membranes but they nevertheless use sophisticated mechanisms resulting in precise intracellular localization of chromosome regions, plasmids, proteins, and RNA [214–217]. Despite a completely different architecture of their principal nucleases, the degradation machineries of both *E. coli* and *B. subtilis* are essentially localized at the cell periphery. The *E. coli* degradosome is localized to the cytoplasmic membrane [218–220] and this localization is important for normal growth [218]. RNase E is tethered to the inner membrane via a short amphipathic helix present at the beginning of the C-terminal half of RNase E (residues 565–582 in *E. coli* RNase E) and which is conserved in the β- and γ-proteobacteria [218]. In addition, in vitro the catalytic domain of RNase E may associate with membrane phospholipids through electrostatic attraction and this affects ribonuclease activity by stabilizing the protein fold [219]. The finding that RNase E as well as RhlB are components of a helical cytoskeletal structure [220, 221] have been subject to debate, especially since the existence of a bacterial cytoskeleton is poorly supported by recent work [222–224].

*B. subtilis* RNase Y has an N-terminal transmembrane domain and is found associated with the membrane [166] (the *rny* gene is named *ymdA* in this reference). It has been identified as one of three proteins that likely interact with the bacterial dynamin-like protein DynA. This interaction may contribute to the correct localization of RNase Y [225]. The similar sublocalization of RNase E and RNase Y, two enzymes with completely different primary sequences, but which are functionally related, clearly shows the importance of this compartmentalization in RNA metabolism. However, it is equally important to know where mRNAs are localized in the cell and how they will eventually meet the enzymes that will destroy them. In *B. subtilis*, transcription and translation have been shown to occur predominantly in separate functional domains with ribosomes distributed around the cell periphery and particularly concentrated at the cell poles [226, 227]. Super-resolution imaging in live *E. coli* cells gives a very similar picture [228, 229]. Nucleoid-ribosome segregation is strong, 85–90 % of ribosomes are some 300–500 nm away from DNA. This suggests that most translation occurs on mRNA transcripts that have diffused into the ribosome rich regions [228]. This apparently contradicts the long-standing view that transcription and translation are tightly coupled in *E. coli* as observed early in electron microscopy studies [230]. However, it is possible that the 10–15 % of ribosomes that co-localize with the nucleoid are involved in co-transcriptional translation [228], which may limit their diffusion out of the nucleoid. Likewise, the nascent polypeptides may begin to oligomerize or interact with other macromolecules causing a specific mRNA to be retained near its site of transcription [215]. Therefore, co-transcriptional translation and strong nucleoid-ribosome segregation need not be exclusive but are two phenomena that can co-exist in the cell. In this sense, the recent observation that the bulk of *lacZ* mRNA in *E. coli* remains close to the nucleoid [231] might simply not be representative for other mRNAs.

Now, if the bulk of full-length mRNAs diffuses into the ribosome-rich periphery, perhaps already bound to ribosomes or protected by cold-shock proteins on their way out of the nucleoid [232] then it makes good sense that the mRNA degradation machinery is localized at the membrane. A priori, a low concentration of decay-initiating RNases, in or near the nucleoid, avoids potentially premature and wasteful degradation of those transcripts that normally diffuse to the translation compartment at the periphery where they remain for the longest part of their life-time. Secondly, it allows the actively translated mRNAs to be in close vicinity to the RNase E degradosome. This is probably the biologically most relevant place to be because it permits the degradosome to monitor suboptimal translation and initiate mRNA decay in order to maintain the efficiency of gene expression. Interestingly, in *E. coli* the major 3′ exoribonuclease RNase II also localizes to the membrane via an N-terminal amphipathic helix and its membrane tethering is important for maintaining cell viability in the absence of PNPase [233]. Thus, there appears to be a similar spatial separation of the transcription, translation and RNA degradation machineries in the two very distant bacteria *E. coli* and *B. subtilis.*

This might nevertheless not be a universally conserved feature in bacteria. In the α-proteobacterium *Caulobacter crescentus*, mRNAs co-localize with their cognate genes for extended periods of time and most ribosomes appear tethered to the DNA via the translated mRNA in live imaging experiments [231]. In addition, chromosomal DNA and ribosomes are distributed homogeneously in the cytoplasm, leaving mRNAs in close proximity to ribosomes without the need to diffuse to different regions of the cell [231]. The *Caulobacter* *E. coli*-type RNase E-based degradosome, whose abundance varies through the cell cycle [205], is not tethered to the membrane but associated with the DNA [231]. This chromosome-centric organization is fundamentally different from that observed in *E. coli* and *B. subtilis.* It represents a coherent alternative model in which transcription, translation and initiation of mRNA decay may be organized using the chromosomal layout as a template. Whether this configuration could be advantageous for an asymmetrically dividing organism like *C. crescentus* is not clear. RNase R, which is thought to play a role in the turnover of tmRNA involved in ribosome rescue, is found tethered to the membrane in *C. crescentus* [234]. This suggests that segregation of some RNA decay enzymes to the periphery is also used in this organism.

Finally, we would like to point out that the localization of a decay-initiating RNase at the membrane might not be synonymous with the obligation for the substrate RNA to diffuse to the cell periphery in order to be cleaved. In this respect, an unrelated but relevant observation made with a membrane bound Lac repressor is pertinent, at least in *E. coli.* Indeed, the Lac repressor whether artificially attached to the cytoplasmic membrane or in its freely diffusible form finds its operator site in the chromosome with similar efficiency [235]. Even more relevant to the current topic, a membrane-bound transcriptional antiterminator protein (i.e., the *E. coli* BglG protein) is capable of interacting fast enough with its chromosomally encoded nascent mRNA target sequence to promote transcriptional read-through [236]. Recently, visualization of living *E. coli* nucleoids have revealed a dynamic helical structure with a high internal mobility, up to 10 % of nucleoid density can shift back and forth in waves within 5 s [237]. *B. subtilis* nucleoids exhibit a similar helical shape [238]. The components of the nucleoid, be it DNA or RNA, are thus mobile and seem able to gain rapid access to the inner membrane. If this turns out to hold true, then the apparent incompatibility between co-transcriptional degradation [239] and the membrane location of the major RNases does not exist anymore.

## Regulated mRNA decay

Modulating RNA decay is a very efficient means to adjust the levels of gene expression. It is thus not surprising that many diverse and intricate mechanisms have evolved to use RNA degradation as a post-transcriptional control of gene expression. In a very direct way, ribonucleases often exploit their activity to feedback regulate their own expression by adjusting cognate mRNA decay. This is notably the case for three ribonuclease genes in *E. coli*, *rne* (RNase E) [153, 240], *pnp* (PNPase) [241, 242] and *rnc* (RNase III) [38, 243], the latter is also autoregulated in *Streptomyces coelicolor* [244]. Less directly, the timing and means of the decay-initiating event can be modulated by proteins that alter the behavior of a ribonuclease, by small RNAs that “indicate” the nuclease where to cleave or serve as an anchor to bind an adapter protein, and by riboswitches that become ribozymes. These specific regulatory mechanisms can regulate the decay of a single RNA but also influence the abundance of hundreds of transcripts, often in response to a specific metabolic or stress condition. Here we will give a short overview illustrating the diverse possibilities to tune mRNA degradation.

### Modulators of RNase E activity

The activity of RNase E can be modulated by a number of regulatory proteins that directly interact with various regions of the enzyme. Two of them RraA (regulator of ribonuclease activity A) and RraB can repress RNase E activity affecting the abundance of several hundred mRNAs but they require significant overexpression to produce an observable effect in vivo [245]. Deletion of RraA caused the destabilization of ~80 transcripts but did not affect growth [245]. Both regulators bind to the RNase E CTH and to the C terminus of RhlB [246] and RraA is induced upon entry into stationary phase [247]. The full physiological role of RraA and RraB (i.e., when not overexpressed) remains to be determined. A similar effect has been described for ribosomal protein L4 that can bind to the CTH of RNase E and alter the abundance of dozens of mRNAs when ectopically expressed [199]. L4 has been proposed to be released by ribosome degradation during starvation [197] and may contribute to the high expression of stress proteins under adverse conditions [199].

A protein interacting with RNase E can also allow a very specific control of a single RNA. The small RNA GlmZ is required to activate the target *glmS* mRNA expression by base-pairing. The direct interaction of RapZ (YhbJ), a novel type of RNA binding protein, with the catalytic domain of RNase E helps recruit the nuclease to the GlmZ RNA. Cleavage of GlmZ removes the region required for activation. The adaptor function of RapZ is further regulated by GlmY a sRNA similar to GlmZ that can bind RapZ competitively with GlmY and inhibit the recruitment of RNase E to GlmZ [248].

### Small RNAs

Currently, small *trans*-encoded regulatory RNAs constitute one of the most dynamic fields in prokaryotic research. They regulate mRNA activity by short, imperfect base-pairing interactions that generally occur at or around the RBS of an mRNA target [249, 250]. This basepairing interferes with ribosome loading and was initially thought to explain reduced mRNA stability. However, recent studies show that mRNA decay initiated by RNase E or RNase III can be directly regulated by sRNAs [251]. In Gram-negative organisms, the RNA binding protein Hfq plays an important role for the function and/or stability of this family of sRNAs [252]. The major RNase implicated in this pathway is RNase E and its scaffolding C-terminal domain is often required for the sRNA-mediated response. Deletion of this portion of the enzyme weakens sRNA-induced silencing of some target genes [253–255] as well as the degradation of some sRNAs [256]. The decay of the sRNA can be coupled or uncoupled to that of the target mRNA [253, 257, 258]. Polynucleotide phosphorylase, a 3′–5′ exonuclease has an important role in the degradation of sRNAs which are not bound to Hfq in stationary phase [259]. This enzyme was previously shown to also have a positive effect on the level of certain mRNAs, possibly through its action on small RNA [260]. Consistent with this idea, PNPase protects some sRNAs from premature degradation by RNase E during exponential growth [9, 259, 261]. The details of how PNPase in conjunction with Hfq and RNase E affects sRNA trafficking and the fate of the target mRNAs remain to be explored.

It has been proposed that the CTH of RNase E actually recruits the Hfq: sRNA complex [150, 196] and binding studies suggest that RNA can bridge between Hfq and the RNA binding domains located in the CTH of RNase E [262]. Guidance of RNase E to the target mRNA by the sRNA has thus some resemblance to the action of microRNA mediating eukaryotic RISC. Alternatively, the initial Hfq stimulated sRNA-mRNA interaction could take place independently of RNase E. This might be the case when the 5′ monophosphorylated MicC sRNA pairs with the *ompD* target mRNA. The monophosphate at the 5′ end probably tethers RNase E and stimulates cleavage of the *ompD* mRNA [257]. Interestingly, the site of cleavage by RNase E can be adjacent to the sRNA-mRNA duplex as in the case of *ompD* [254] but also distant from the pairing region [255]. The molecular determinants for binding and cleavage by RNase E remain ambiguous. RNase E can recognize and bind a 5′ P but possibly also recognizes the sRNA-mRNA duplex in a similar manner when it binds to a secondary structure in autoregulatory mode [263].

The recent finding that RNase E may bind to polysomes [264] opens new perspectives of how RNase E may operate also in sRNA-mediated decay. It has been proposed that while interacting with polysomes the degradosome might remain associated with the Hfq:sRNA:mRNA complex formed at or near the RBS. The mRNA emerging from the last translating ribosome could at some point contain a structural signal recognized by the degradosome and provoke cleavage [265]. Direct interaction with the ribosome might also enable RNase E to increase the probability of cleaving an appropriate site between translating ribosomes independent of sRNA mediation (direct entry pathway).

Documented sRNA initiated mRNA decay is still rare in Gram-positive bacteria [266]. Probably the best characterized system involves the *S. aureus* regulatory RNAIII [267] that is involved in the control of virulence by repressing the expression of adhesin factors and the transcriptional regulator Rot [268, 269]. Base-pairing of RNAIII with several mRNAs, independently of Hfq, is quite extensive and can trigger initiation of decay by RNase III by exploiting its propensity to cleave uninterrupted RNA duplexes [270]. In *Listeria*, the LhrA sRNA with the help of Hfq modifies the expression of almost 300 genes and in two cases has been shown to interact with the mRNAs, repressing translation and inducing their degradation by a yet unidentified ribonuclease [271, 272].

In *B. subtilis,* SR1 sRNA is expressed under gluconeogenic conditions. It can inhibit translation of *ahrC*, a transcriptional regulator of arginine catabolic operons through base-pairing [273]. However, SR1 can also inhibit degradation of the *gapA* operon mRNA which encodes glycolytic enzymes. Interestingly, this SR1 action does not depend on base-pairing but instead requires the small peptide encoded by the sRNA, called SR1P. SR1P interacts with the GapA protein (glyceraldehyd-3-phosphate dehydrogenase) but the mechanism by which stabilization of the *gapA* operon mRNA is achieved remains obscure [274]. Direct interaction between SR1P and GapA may inhibit the reported ribonucleolytic activity of GapA [275]. Both functions of SR1 are conserved in *Bacilli* [276].

### Riboswitches and ribozymes

Across different bacterial *phyla*, riboswitches are being used extensively to control gene expression by directly sensing the concentration of a metabolite [277, 278]. Many of them regulate premature transcription termination, while others regulate translation initiation by sequestering the RBS. The latter functionally inactivates the mRNA, generally causing rapid decay of the transcript. However, metabolite binding can also directly initiate the degradation of an mRNA. A very pertinent example is the *B. subtilis glmS* gene encoding glucosamine-6-phosphate (GlcN6P) synthase. When in excess, GlcN6P binds to the 5′ UTR and induces autocatalytic cleavage at a 5′ proximal site leaving a 5′ OH group on the downstream fragment containing the *glmS* open reading frame [279]. This creates an entry site for the 5′ exonuclease activity of RNase J1 that rapidly degrades the mRNA [117]. Interestingly, the *glmS* riboswitch-ribozyme is also the first example that defies the conventional view that a riboswitch recognizes a single cognate metabolite. It can actually integrate information from an array of hexose metabolites to both activate and inhibit self-cleavage. Initiation of mRNA decay is thus used to assess the overall metabolic state of a cell [280].

In *E. coli*, a lysine responsive riboswitch controls translation initiation of the *lysC* mRNA. However, in the absence of lysine, the riboswitch adopts a conformation that not only liberates the RBS for ribosome binding but also sequesters RNase E cleavage sites. When bound to lysine, RNase E cleavage of the now accessible sites in the 5′ UTR contributes significantly, and independently of translation inhibition to the decay of the *lysC* transcript, rendering repression definitive [281].

Similarly, in *B. subtilis*, RNase Y cleavage in the *yitJ* SAM riboswitch only occurs when SAM is bound to the aptamer. However, since the *Bacillus* SAM riboswitch acts transcriptionally the antiterminated full-length mRNA is not cleaved and the primary action of RNase Y, in this case, is the turnover of the riboswitch after termination has occurred [59].

## RNase toxins

Toxin-Antitoxin (TA) systems are an interesting reservoir for novel ribonucleases and are continuously being discovered across all bacterial species. TA modules are not essential for normal cell growth but are probably advantageous for cell survival in their natural habitats [282]. The toxins may allow the cells to adapt to changing environments and increasing persistence (persisters are dormant cells that resist toxic treatment that kill the majority of their siblings) by slowing and inhibiting cell growth or causing some cells to die. They exert their action by targeting essential cellular processes including protein synthesis, DNA replication, cell-wall biosynthesis and mRNA stability [283]. The antitoxins, being unstable compared to the cognate toxins, have to be continuously synthesized to constantly inhibit toxin function [284]. TA modules are currently classified into five types based on the molecular identity of the components and/or the mechanism of action [285]. Ribonucleases notably play a role in regulating type I toxin translation which is turned off by small antisense RNAs that act as antitoxins [286]. For example, in *E. coli* RNase III has been shown to cleave the mRNA-asRNA hybrid in several cases and render the repression of toxin translation irreversible [287, 288]. However, a number of toxins are themselves ribonucleases that cleave cellular mRNA and have been termed RNA interferases [284, 285]. They come in two versions: they either cleave mRNA in a ribosome-dependent way (e.g., RelE) or in the absence of ribosomes (e.g., MazEF, VapBC, and ToxN).

Ribosome-dependent mRNA interferases have no or only very weak endoribonuclease activity by themselves [284]. The RelE component of the RelBE TA system, one of the best studied representatives of this family, is activated during the stringent response [289] and associates with the ribosome A site to induce mRNA cleavage [290, 291]. Upon RelE binding to the ribosome the mRNA in the A site is significantly repositioned leading to 2′-OH-induced hydrolysis [292]. Determination of the cleavage specificity of a variety of heterologous RelE proteins in *E. coli* indicates a preference for cleavage upstream of purines and between the second and third position of codons [293].

In *E. coli,* MazF has been proposed to mediate programmed cell death under a variety of conditions, including the presence of DNA damaging agents, nutrient starvation, phage infection, high temperature and antibiotics [294, 295]. It is a sequence-specific endoribonuclease that produces a 2′,3′-cyclic phosphate on the upstream cleavage product and a free 5′-OH group on the downstream fragment [296]. *E. coli* MazF cleaves mRNA at ACA sequences effectively shutting down protein synthesis [297]. This capacity has been exploited to convert *E. coli* into a bioreactor producing only the target protein from an engineered mRNA devoid of ACA sequences [298]. A large number of MazF homologs have been identified in bacteria and in Archaea [282]. Interestingly, these variants cleave mRNAs with varying recognition sequences comprising three, five or seven bases with the cleavage site extending mostly at the 3′ end [299]. For example, the MazF homologue EndoA (*ndoAI/ndoA* module) in *B. subtilis* cleaves at unpaired UACAU sequences [300]. As the recognition sequences become longer the target mRNAs become fewer and the RNA interferase can effectively silence gene expression specifically in cells [299]. However, even the short ACA recognition site of *E. coli* MazF can provide specificity. Cleavage of ACA sites at or immediately upstream of the AUG start codon of specific transcripts generates leaderless mRNAs. At the same time, removal of the anti-Shine and Dalgarno sequence on the 16S rRNA by MazF creates a subpopulation of ribosomes capable of translating leaderless mRNAs [301]. Thus, stress-induced expression of MazF (e.g., during the stringent response) can result in the adaptation of the translation machinery leading to selective translation of certain mRNAs in response to the physiological state of the cell.

The VapBC family (*v*irulence *a*ssociated *p*rotein) is the most widely distributed TA module in microbial genomes with up to 47 putative *vapBC* operons found in *M. tuberculosis* [302]. Physiological studies show that VapC plays a key role in adapting bacteria to a variety of growth conditions and new environments [285]. The VapC component belongs to the PIN-domain family of proteins predicted to be Mg^+2^-dependent RNases with an active site architecture similar to phage T4 RNase H and the FLAP endonucleases [303]. The diverse structures of prokaryotic PIN-domain proteins indicate that the groove containing the active site is formed through dimerization of identical subunits [285]. VapC from the enteric bacteria *Shigella* and *Salmonella* can inhibit translation by cleaving tRNA^fMet^ in the anticodon stem loop [304]. The mycobacterial VapC toxin can cleave mRNA in a site-specific manner. The target sequence is -AUA(U/A)-hairpin-G- indicating that the RNA secondary structure is part of the recognition motif. In contrast to the MazF RNA interferase, VapC cleavage products carry a 3′ OH on the upstream fragment and a 5′ monophosphate on the downstream fragment as observed for most classical ribonucleases [305, 306].

ToxIN is the defining member of type III TA systems where an antitoxin RNA binds and inactivates the toxin. It is found in a number of Gram-positive and Gram-negative pathogenic bacteria [307]. ToxN is a sequence-specific endoribonuclease that has a structure similar to MazF and also generates 2′–3′ cyclic phosphate and a 5′-OH on the cleavage products. It preferentially cleaves RNA at AA^A(U/G) (*B. thuringiensis*) and A^AAAA (*P. atrosepticum*) sequences [308, 309]. ToxIN can also act as an abortive infection (Abi) system where a phage-infected bacterium, that is no longer able to synthesize the antitoxic RNA, is prematurely killed to prevent the release of viral progeny and protect the wider population. It is at present the only TA/Abi system that can protect multiple bacterial genera against different phages [307].

In the recently discovered TA system GhoST, (now referred to as type V) in *E. coli* and *Shigella,* the antitoxin GhoS is not labile during stress. It is a sequence-specific endoribonuclease that specifically cleaves the *ghoT* toxin mRNA in A/U rich regions. GhoS adopts a ferredoxin-like fold that is very similar to the Cas CRISPR RNases [310]. Interestingly, despite a rather undefined recognition sequence, forced expression of GhoS reduced the abundance of only 20 transcripts, all of which were involved in the biosynthesis/transport of purines and pyrimidines [311]. GhoTS is the first TA system to be regulated by another TA system, MqsRA. During stress the MqsR endoribonuclease preferentially degrades the antitoxin GhoS mRNA over toxin GhoT mRNA, yielding free toxin [312].

## Conclusions

Recent progress in deciphering the components and pathways involved in mRNA metabolism in a variety of organisms clearly supports the pervasive idea that of low-specificity endonucleases are important for initiating bacterial mRNA decay. The conservation of a vague but similar endonucleolytic cleavage specificity for the three major decay-initiating ribonucleases E, J and Y constitutes an impressive case of convergent evolution. The preference for a 5′ monophosphorylated RNA substrate is another feature shared by these structurally unrelated enzymes. In fact, a 5′ P moiety is required for the 5′ exonuclease activity of RNase J, and for stimulating the endonucleolytic activity of RNases E and Y, at least in vitro. This similarity again illustrates the power of convergent evolution to develop key biological functions. One of the reasons why some organisms rely on a 5′ exoribonuclease, generally occurring together with RNase E or RNase Y might be linked to the presence or absence of an effective polyadenylation-assisted degradation pathway for 3′ structured RNA fragments. Indeed, the only way to get rid of fragments protected against 3′ exonuclease attack is to degrade them from the other side.

Multi-protein degradosome complexes are efficient machineries to streamline the degradation process. Even though they probably exist in all bacteria, they vary greatly in their composition and the importance of the proposed interactions in RNA decay in vivo remains to be elucidated. Degradosomes based on protein–protein interactions are clearly important but maybe they will turn out to be only a part of the potential complexes that might exist. The capacity of small RNAs to alter enzyme specificity by serving as a scaffold to bring together proteins of diverse activities opens up completely new possibilities of adapting mRNA metabolism to varying physiological conditions.

Last but not least, spatial organization of transcription, translation and mRNA decay could have a profound influence on how mRNA decay affects gene expression. In this respect, future studies should not only look at the functional importance of the membrane localization of major ribonucleases but also at the dynamics of the nucleoid and the nascent transcripts.

## Abbreviations

NTH: N-terminal half
CTH: C-terminal half
RBS: Ribosome binding site
